# Evolutionary History of Trihelix Family and Their Functional Diversification

**DOI:** 10.1093/dnares/dsu016

**Published:** 2014-05-25

**Authors:** Yao Qin, Xin Ma, Guanghui Yu, Qi Wang, Liang Wang, Lingrang Kong, Wook Kim, Hong Wei Wang

**Affiliations:** 1 State Key Laboratory of Crop Biology, College of Agronomy, Shandong Agricultural University, 61 Daizong Street, Tai'an, Shandong 271018, People's Republic of China; 2 Division of Biotechnology, College of Life Sciences and Biotechnology, Korea University, Seoul 136-713, Republic of Korea; 3 Agronomy College, Sichuan Agricultural University, No. 211, Huiming Road, Wenjiang Region, Chengdu 611130, PR China

**Keywords:** trihelix, abiotic stress, sorghum, subfunctionalization

## Abstract

In this study, we carried out an evolutionary, transcriptional, and functional analyses of the trihelix transcription factor family. A total of 319 trihelix members, identified from 11 land plant species, were classified into five clades. The results of phylogeny indicate the binding domains of GT1 and GT2 diverged early in the existence of land plants. Genomic localization revealed that the trihelix family members were highly conserved among cereal species, even though some homeologs generated during the tetraploidy of maize were lost. Three-dimensional structural analyses and an examination of subcellular localization of this family supported the involvement of all five clades in transcriptional regulation. Furthermore, the family members from all clades in sorghum and rice showed a broad and dynamic expression pattern in response to abiotic stresses, indicating regulatory subfunctionalization of their original functions. This finding is further supported by the phenotypes of enhanced tolerance to cold, salt, and drought in transgenic plants overexpressing Sb06g023980 and Sb06g024110. In contrast, few *Arobidopsis* genes showed inducible expression under abiotic stress conditions, which may indicate a functional shift. Finally, our co-expression analysis points to the involvement of this family in various metabolic processes, implying their further functional divergence.

## 1. Introduction

Transcriptional regulation of gene expression plays a major role in both plant development and in response to environmental stimuli. Each is controlled by various classes of transcriptional factors (TFs), either by interaction with *cis*-acting elements, or with other TFs required for gene expression.^1,2^ In plants, there are more than 60 TF families currently identified with varied functional roles that are being progressively defined.^1,2^ The trihelix family, one of the first TFs discovered in plants, is classified as GT factors due to their binding specificity for GT elements.^3–6^ The DNA-binding domain of GT factors features a typical trihelix (helix-loop-helix-loop-helix) structure. Taken together, the helices form a bundle held together by a hydrophobic core that determines the specific binding of GT elements with a degenerate core sequence of 5′-G-Pu-(T/A)-A-A-(T/A)-3′.^4,6–8^

The first trihelix TF of GT-1 was isolated from pea (*Pisum sativum*). It binds to the promoter region of the *rbcS-3A* gene to regulate light-dependent expression.^4^ Some homologous members of the GT-1 family were later found in *Arabidopsis*, rice, and tobacco; but their physiological roles in the light response were still not uncovered.^9–13^ It is notable that the transcription of GT-1 has been found to occur independent of light, which was proposed to transmit signal to their targets through the phosphorylation of a threonine in the trihelix domain.^8,14^

In the last decade, a dozen trihelix genes from various plants were cloned and characterized. They showed a large functional divergence, in processes such as: seed scattering during crop domestication, embryo development, morphogenesis control of manifold flower organs, and biotic and abiotic stresses resistance, etc.^11,15–22^ Most of the functions involved in plant development have been studied in *Arabidopsis*. The *Arabidopsis* transcription factor PETAL LOSS (*PTL*) was reported to function in the morphogenic control of manifold flower organs, such as the perianth, petals, and stamens.^17,23^ Several genes were reported to play divergent roles during the process of whole seed development.^15,18,20^ The knockout mutant (*EDA31*), a close member of *PTL*, was found to be involved in embryo sac development, as it blocked the development before fusion of polar nuclei.^24^ Another gene within the GT-1 clade, present in *Arabidopsis* (At5g63420), encodes a metallo-β-lactamase-trihelix chimera that is abundantly expressed in seeds and has been proved an early embryogenesis requirement. The mutants *asil1* and *asil2* of another two GT factors, *ASIL1* and *ASIL2*, have been shown to accumulate chlorophyll only at the late state of embryo development, which negatively regulates the albumin gene *2S3*, as well as several other seed maturation genes in *Arabidopsis.*^25^ Recently, an important GT factor gene, *SHA1*, was characterized and shown to regulate the seed scattering process during domestication.^19^ This gene was identified by genetic variant and map-based cloning in rice, wild type of which can promote the function of the abscission layer in the pedicel of mature seeds.^26^ Although the gene members across the entire trihelix family participate in plant developmental programmes and light response, two recent studies suggest that some GT factors are also involved in the basic resistance of plants to abiotic stresses, especially salt tolerance.^21,22^ Overexpression of the GT-2-like soybean gene, *GmGT-2B*, was shown to increase tolerance to salt, drought, and freezing, while another gene, *OsGTγ-1*, originally found in *γ* clade, regulated salt resistance when using a reduced expression mutant and overexpressed transgenic lines.^21,22^

While functional knowledge of TFs is still very limited, the currently characterized family members show an immense functional divergence. It is not known whether these functions share common underlying mechanisms. For example, the repression of growth of trichomes,^16^ inter-sepal zones,^17^ and the accumulation of storage products, except during late embryogenesis, may be regulated by similar molecular mechanisms.^18,25^ It will be an important step forward to establish functional relationships between the trihelix family members. There have been 30 gene members identified in *Arabidopsis* and rice; however, a more thorough systematic analysis is needed to uncover the evolutionary and functional information of this family.^11,27^ In this study, we investigated the evolutionary history, expression patterns, and transgenic lines of trihelix genes in *planta* for functional clues. A total of 12 available whole genomes were employed to identify gene family members and to evaluate phylogenetic relationships, genomic loci, subcellular localization, and structural characteristics. In order to further discern gene functions of this family, we combined the analysis with microarray data and semi-quantitative reverse transcription–polymerase chain reaction (RT–PCR) in response to various abiotic stress conditions. Two sorghum trihelix genes were further selected to be cloned and overexpressed in *Arabidopsis* for functional evaluation.

## 2. Materials and methods

### 2.1. Database search and sequence retrieval

Full genome assemblies of 12 plant species representing eudicots (*Arabidopsis thaliana*), magnoliid dicots (*Aquilegia coerulea* and *Mimulus guttatus*), monocots (*Brachypodium distachyon*, *Oryza sativa*, *Setaria italica*, *Zea mays*, and *Sorghum bicolor*), ferns (*Selaginella moellendorffii*), mosses (*Physcomitrella patens*), and algae (*Chlamydomonas reinhardtii* and *Ostreococcus lincimarinus*) were downloaded from the Joint Genome Institute plant genomics database (http://www.Phytozome.net). The expressed sequence tag (EST) sequences of wheat were downloaded from NCBI (National Center for Biotechnology Information). The amino sequences of the trihelix domain were extracted from known members of putative trihelix genes in *Nicotiana tabacum*, *A. Thaliana*, and *O. sativa*, as described in PlnTFDB,^1^ which were used as a query for blast with a cut-off value of *e*^−10^. As for confirmation of the predicted genes, manual correction was performed using the online web server FGENESH (http://linux1.softberry.com/berry.phtml).^28^ The confirmed sequences were further subjected to verify the presence of the trihelix domain using conserved domain analysis.^29^

### 2.2. Phylogenetic analysis

Multiple sequence alignments of the identified trihelix genes were performed by the Clustal W program using default parameters.^30^ To evaluate the fit of major models of amino acid substitutions, the Bayesian information criterion (BIC) and Akaike information criterion (AIC) were applied to select the fit model that was followed by amino acid frequencies and rates of amino acid substitutions for each amino acid pair using discrete gamma distribution. The phylogenetic tree was constructed with the Molecular Evolution Genetic Analysis (MEGA) 5.0 software according to the fit model using the maximum-likelihood (ML) method and the bootstrap test was carried out with 1000 iterations.^31^ To ensure that the more divergent domains could contribute to the topology of the ML tree, all positions with <95% site coverage were eliminated.

### 2.3. *In silico* microarray profiling and co-expression analysis

The expression data of *Arabidopsis* and rice trihelix genes were subjected to an online web-tool, Genevestigator (https://www.genevestigator.ethz.ch), against an Affymetrix platform using default parameters. The expression patterns of trihelix genes in particular organs and at specific growth conditions were presented as heat maps, in which the colour intensity corresponded to the expression level. Genes without probes and those of low quality were not generated for further study. For co-expression analysis, microarray CEL files of *O. sativa* were downloaded from GEO (http://www.ncbi.nlm.nih.gov/geo/). The microarray probe data were retrieved from Affymetirx. The robust multichip average method, provided by Affymetrix power tools (APTs), was used to convert Affymetrix probe level data into expression values.^32^ The Pearson correlation coefficient of two genes was calculated based on each Affymetrix microarray dataset after filtering out low-quality slides. In order to choose an appropriate cut-off value for co-expression gene network construction, we examined the distribution of random pairs, resulting in *r* = 0.585 as a positive co-expression relationship, and *r* = −0.465 as a negative co-expression relationship.^33^ The selected *OsTrihelix* genes from different sub-clades throughout phylogenetic analysis and their co-expressed genes were subjected to the Cytoscape program to visualize the co-expression gene network under edge-weighted force-directed layout.^34^

### 2.4. Chromosomal localization and domain structure prediction

Syntenic gene datasets against chromosome regions among rice, brachypodium, maize, and sorghum were downloaded from PGDB (http://chinna.agtec.uga/edu/duplication). The pan-grass syntenic gene sets were downloaded from the CoGe (http://synteney.cnr.berkerley.edu/CoGe/). The selected collinear trihelix genes were visualized by the Circos program.^35^

The crystal structure of the DNA-binding domain of *Arabidopsis* GT1 (Protein Data Bank code number 2JMW) was used as a template for constructing the structure models of the trihelix protein in each clade according to the phylogenetic analysis. Sequences from each clade were aligned by the Align 2D structure alignment program (homology module, InsightII; Accelrys), respectively. Structures were automatically built by the MODELER module of InsightII. MODELER uses a spatial restraint method to build a three-dimensional image of protein structure and is capable of generating a reliable predicted structure using probability density functions derived from homologous structures and general features of known proteins. Then, molecular dynamics simulations were carried out for the entire system to optimize all protein structures by the GROMACS 3.0 software.

### 2.5. Abiotic stress treatments and RT–PCR

Seedlings of the rice and sorghum were allowed to grow on mesh supported in plastic containers with Murashige and Skoog (MS) solution (16/8-h light/dark photoperiod, at 25°C, with 70% relative humidity) for 14 days.^36^ For abiotic stress treatments, seedlings were treated with salinity (250 mM NaCl), dehydration (25% of PEG 10,000), and cold (4°C). Leaves of five or more seedlings were harvested at 9 h, 1, 2 and 3 days after initiation of the treatments, frozen immediately in liquid nitrogen, and then stored at −80°C. Total RNA was extracted with the RNeasy plant mini kit (Qiagen, Hilden, Germany) according to manufacturer's instructions. The first-strand cDNA synthesis was conducted using a first-strand cDNA synthesis kit for RT–PCR (Roche Diagnostics GmbH, Mannheim, Germany). Semi-quantitative RT–PCR was then conducted as described previously.^37^ A total of 20 gene-specific primer pairs for rice and sorghum trihelix genes were designed. The actin gene was employed as an internal control (Supplementary Table S1).

### 2.6. Subcellular localization

The coding regions of Sb06g023980 (Clade I), Sb06g020670 (Clade II), Sb04g022190 (Clade III), Sb04g004960 (Clade IV), and Sb06g024110 (Clade V) were amplified and cloned into the pBIN35S:EGFP vector, which were transformed into *Agrobacterium* of GV3101. For transient expression of the fusion proteins in *Nicotiana benthamiana*, the resultant *Agrobacterium* culture was resuspended in infiltration medium (10 mM 4-morpholineethanesulfonic acid hydrate, pH 5.6, 10 mM MgCl_2_, and 200 mM acetosyringone) and then injected into 4-week-old *N. benthamiana* leaves with an optical density of 0.5 OD at 600 nm. The addition of transformed 35S:EGFP *Agrobacterium* was used as a control. Confocal microscopy was used to assess the results at 3 days post-*N. benthamiana* leaf infection. Fluorescent images were obtained using an LSM 510 META NLO system (Carl Zeiss, Oberkochen, Germany).

### 2.7. Transgenic *Arabidopsis* analysis

*Agrobacterium* strains GV3101 containing pBIN35S:Sb06g023980 and pBIN35S:Sb07g02907 were used to transform *Arabidopsis* plants according to the floral-dip method.^38^ Transgenic lines were selected on MS agar plates containing 30 mg/l kanamycin and the T_3_ lines were used for further phenotypic analysis. Two-independent Sb06g023980 and Sb07g02907 overexpressing *Arabidopsis* lines were tested to observe the effects of cold, salt, and drought stresses. The transgenic seeds were germinated on 1/2 MS medium for 2 days and transferred into medium containing 75, 125, 150, and 180 mM NaCl for the salt tolerance test and moved to 4°C for cold tolerance analysis. Plant growth was then monitored and photographed after 14 days. For drought treatment, the wild-type and transgenic plants were grown in soil for 3 weeks, after which water was withheld for phenotype observations.

## 3. Results

### 3.1. Trihelix family in *planta*

**Figure 1.**
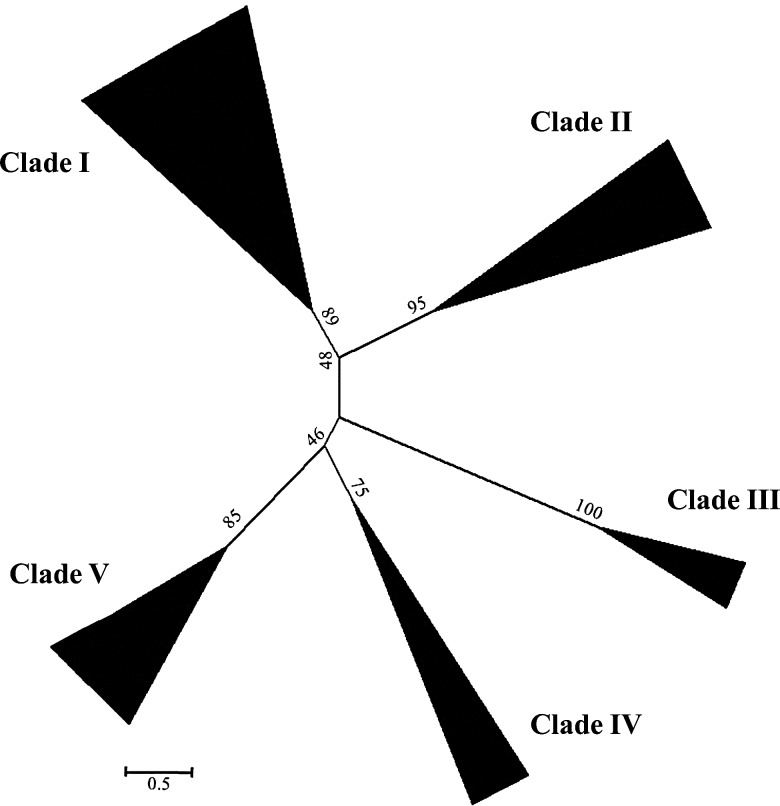
Phylogenetic relationship among trihelix gene members from the studied species of land plants. The evolutionary history was inferred using the ML method. The bootstrap consensus tree, inferred from 1000 replicates, is taken to represent the evolutionary history of the taxa analysed. Branches corresponding to partitions reproduced in less than 95% bootstrap replicates are collapsed. The evolutionary distances were computed using the number of differences method and are in the units of the number of amino acid differences per sequence.

Full genome sequences and ESTs from the algae *C. reinhardtii* and *O. lincimarinus*, the moss *P. patens*, the fern *S. moellendorffii*, the grass *B. distachyon*, *O. sativa*, *S. italica*, *Z. mays*, *S. bicolor*, and *T. aestivum,* the magnoliid dicot *A. coerulea* and *M. guttatus*, and the eudicot *A. thaliana* were blasted for trihelix genes with the putative trihelix-binding domain as a query. Initially, a total of 319 non-redundant putative trihelix genes were identified: 29 in *P. patens*, 25 in *S. moellendorffii*, 28 in *B. distachyon*, 30 in *O. sativa*, 39 in *S. italica*, 48 in *Z. mays*, 27 in *S. bicolor* and 8 in *T. aestivum*, 27 in *A. coerulea*, 28 in *M. guttatus*, and 30 in *A. thaliana*. However, no trihelix genes were found in algae. The identified trihelix genes showed divergent molecular characteristics with protein lengths ranging from 210 to 1045 amino acids, molecular weights ranging from 23.06 to 114.53 kDa, and isoeletric points ranging from 4.51 to 10.02 (Supplementary Table S2–S6). To construct a phylogenetic tree, we first performed a model test among the putative trihelix proteins using an ML procedure.^3,39^ The models with the lowest AIC and BIC scores were considered to best describe the substitution pattern.^40^ In total, 48 different amino acid substitution models were tested by the MEGA 5.2.2 software using a discrete Gamma distribution. This revealed a Jones–Taylor–Thornton (JTT) evolutionary model with five categories that fitted the evolutionary pattern to construct the ML tree (Fig. 1). The topology of the phylogenetic tree, bootstrap values, and sequence identity (>30%) were used to classify the trihelix genes into five distinct clades. The moss and fern trihelix genes were found in relative rooting positions of Clades I, II, IV, and V, indicating they had independently diverged from ancestral land plants and further expanded from vascular plants and angiosperm (Supplementary Table S2–S6 and Fig. S1). Clade III was the only to contain angiosperm-specific trihelix genes (Supplementary Table S4). All trihelix genes in Clades II, III, IV, and V contain a single trihelix domain; however, the members in Clade I are constituted by both single and double trihelix domains (Supplementary Table S2–S5 and Fig. S2). Evidence did not indicate which domain emerged first; however, the existence of gene members with double domains from moss species indicate that the single or double domain structure of trihelix genes likely diverged early given their emergence in land plants (Supplementary Table S2).

The alignment of the sequences of each clade showed that all the clades contain a conserved tryptophan (W) in each repeat, a typical feature of the trihelix domain (Supplementary Fig. S2). In Clade I, the third α-helix often contained a conserved phenylalanine (F) and cysteine (C) instead of tryptophan. Previous research suggests that a fourth amphipathic α-helix with a conserved mode of (F/Y)-(F/Y)-X-X-(L/I/M)-X-X-(L/I/M) aids the third α-helix for DNA binding. Here, we found that it located closely downstream of the trihelix domain in most members of Clades I, II, III, and V, but not in Clade IV (Supplementary Fig. S2). Another conserved structure was located in the C-terminal half in members of all the clades, which is possibly associated with dimerization of these TFs (Supplementary Fig. S2). This domain seems rich of L and E, but not conserved between each clade.

### 3.2. Genomic organization, structure, and subcellular localization

**Figure 2.**
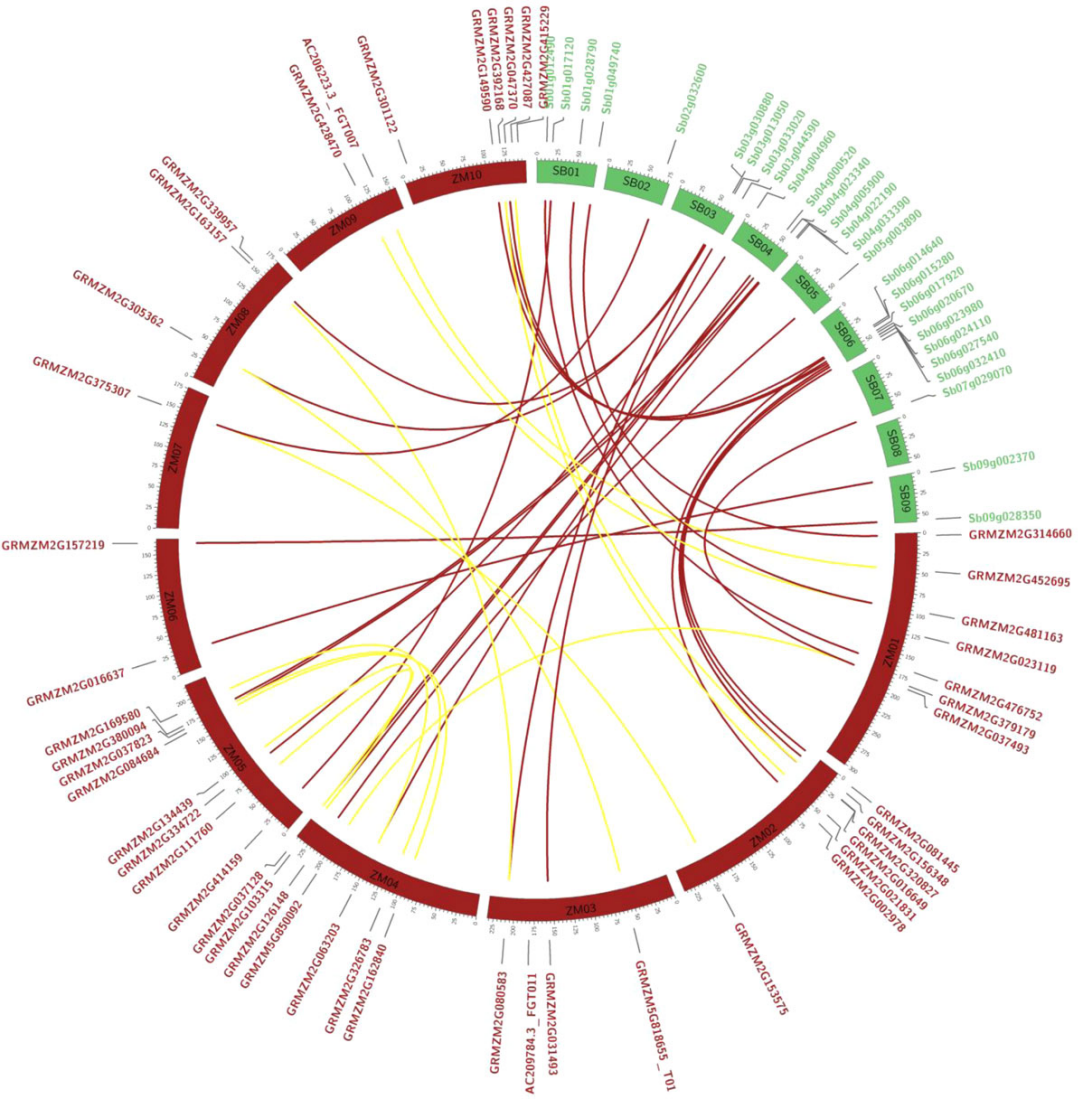
Genomic organization and collinearity of the trihelix family in sorghum and zea maize. The orthologous pairs between sorghum and zea maize are indicated by magenta, and the inter-homeologs of zea maize are indicated by yellow.

To investigate the genomic organization of trihelix genes, duplication blocks were downloaded from the database, as described in Materials and methods. With the exceptions of chromosomes 6 and 7 of *O. sativa* and chromosome 10 of *S. bicolor*, the trihelix genes were evenly distributed on most chromosomes of the tested cereal species (Supplementary Fig. S3). Strong gene conservation was detected in duplication blocks and a collinear relationship with homologs was established for: 29 of 30 genes in *O. sativa*, the entire 28 genes in *S. bicolor*, the entire 28 genes in *B. distachyon*, and 43 of 47 genes in *Z. mays* (Fig. 2 and Supplementary Fig. S3). All the collinear gene pairs in *O. sativa*, *S. bicolor*, *B. distachyon*, and 30 genes in *Z. mays* were true orthologs between each species. A total of 13 collinear gene pairs between different chromosomes were detected in *Z. mays*, which likely resulted from the ancient tetraploidy processes in evolution.^41^ However, four *Z. mays* trihelix genes of GRMZM2G037493, GRMZM2G415229, GRMZM2G023119, and AC209784.3_FGT011 were not found in any duplication blocks, suggesting that there were independent gene duplication events (Fig. 2). The rice trihelix gene of Os03g18330 seems to be a tandem duplication copy of Os03g18340. Although most of the genes were well preserved in the tested cereal species, rare instances of gene loss after duplication were also detected, such as the ortholog of Sb06g015280 in rice, the orthologs of Os02g31160 and Os04g45940 in sorghum, and the ortholog of Os02g43300 in brachypodium (Supplementary Fig. S3).

To obtain the structural characteristics of the trihelix family, the sequences from each clade were aligned by the Align 2D structure alignment program using *Arabidopsis* GT1 as a template and the predicted structures were evaluated.^42^ Generally, the results of protein three-dimensional structure modelling illustrated that the structure of the trihelix factors in each clade was conserved, especially in the N-terminal domain (Supplementary Fig. S4A). Eighty-eight simulated trihelix structures were constructed and five typical structures were selected and superimposed to evaluate the goodness of fit of the overall topologies via Ramachandran plot analysis (Supplementary Fig. S4A and B). The constitution of amino acid residues showed the most favoured with the plots indicating the goodness of fit of selected models. Furthermore, the model within each clade confirmed the same conformation of the structural elements of helices. Structural modifications were present in loop regions of the C-terminal domain and in the linker between the N- and C-terminal domains, where the structural conservation is relatively varied according to the profile scores (Supplementary Fig. S4A and C). The protein sequence differentiation in these regions is high among protein members of each clade. This observation may be due to either relaxed functional constraints or to sequence divergence from the selected template.

**Figure 3.**
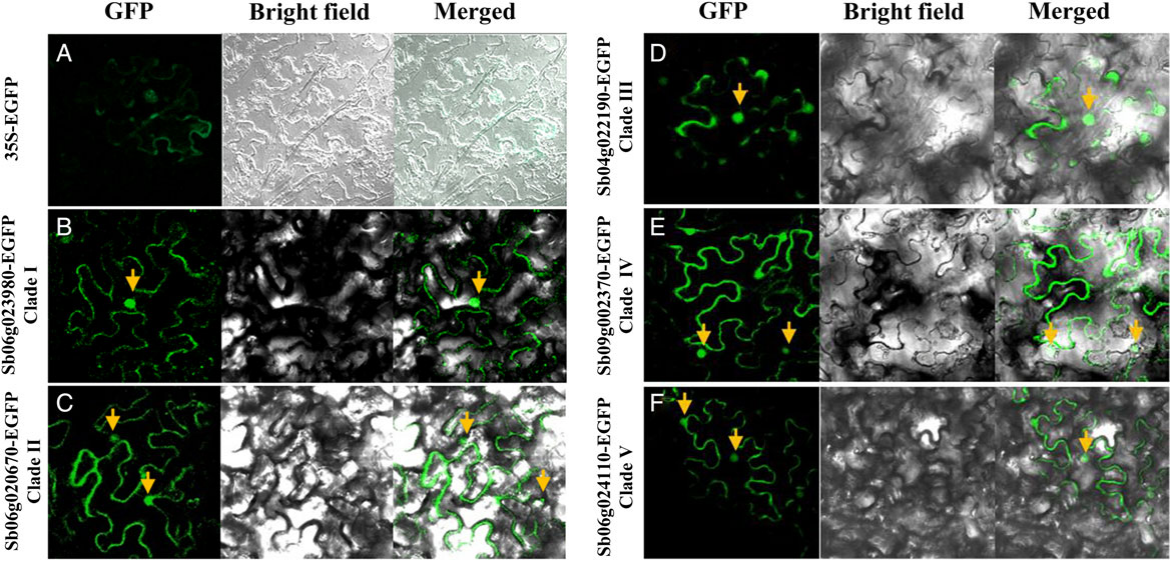
Subcellular localization of trihelix–EGFP fusion proteins in tobacco leaves. *Agrobacterium* strains GV3101 harbouring each construct of 35S:EGFP (A), Sb06g023980-EGFP from Clade I (B), Sb06g020670-EGFP from Clade II (C), Sb04g004960-EGFP from Clade IV (D), Sb04g022190-EGFP from Clade IV (E), and Sb06g024110-EGFP from Clade V (F) were transiently expressed in *Nicotiana* leaves. Images were captured and merged by z-series optical sections after 3 days of agro-infiltration. A 35S:EGFP construct was used as a control.

To further characterize this gene family, the subcellular localization of rice trihelix genes was studied. Five sorghum trihelix genes that are representative of one of the five clades, Sb06g023980, Sb06g020670, Sb04g004960, Sb04g022190, and Sb06g024110, were chosen to construct an EGFP fusion protein under the control of the 35S promoter and transitionally expressed in *N. benthamiana* leaves. The transient expression of 35S:EGFP was observed in both the cytosol and nuclei with a weak signal (Fig. 3A). When fused with the EGFP, all five of the rice trihelix genes directed nuclear expression exclusively, supporting functional roles in transcriptional regulation (Fig. 3B–F).

### 3.3. Transcriptional responses of trihelix genes against abiotic stresses

**Figure 4.**
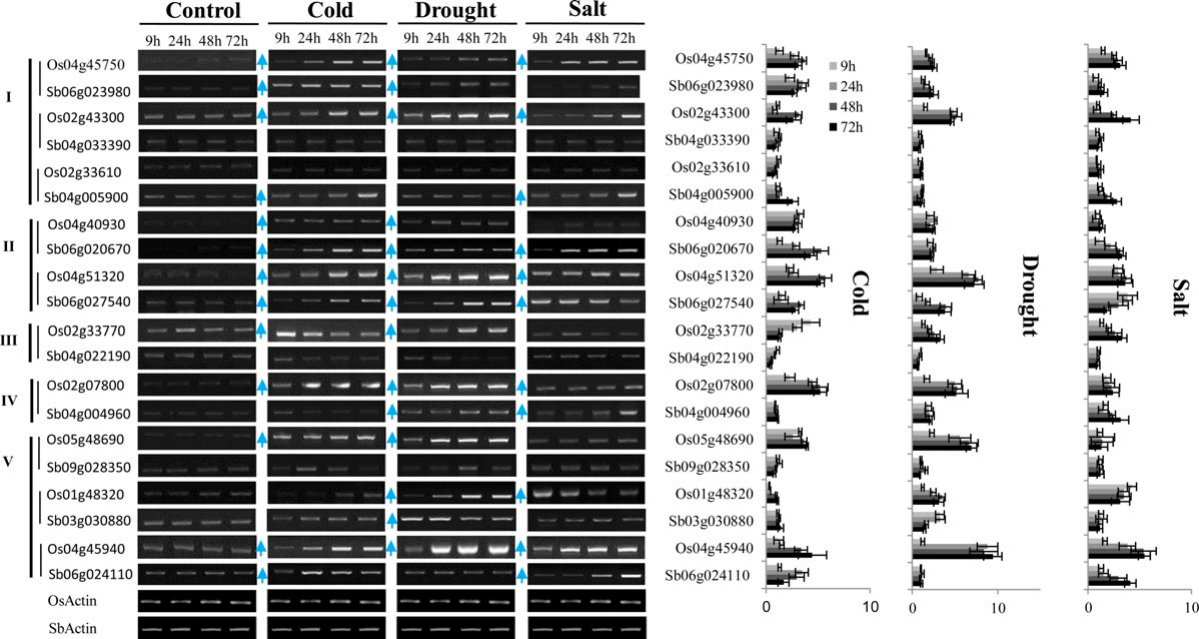
RT–PCR analyses of the expression of trihelix genes against abiotic stress. The stress treatments were applied by subjecting 10-day-old seedlings to drought (polyethylene glycol, 25%), salt (NaCl, 250 mM), or cold (4°C) stress for variable time periods, ranging from 0 to 72 h. (A) Representative semi-quantitative RT–PCR analysis. The collinear trihelix gene pairs are indicated by a vertical line before the gene names. (B) Statistical analysis of the semi-quantitative RT–PCR is shown as mean + SEM for three to four independent experiments.

To investigate expression divergence, we first examined the expression pattern of the trihelix family of rice and *Arabidopsis* using public microarray data. Most of the trihelix genes in *Arabidopsis* were shown to be ubiquitously expressed at tissue-specific level; however, the genes in Clade I seemed to be preferably expressed in flower organs, whereas those in other clades showed a strong signal in seeds (Supplementary Fig. S5). Similar to *Arabidopsis*, most of the rice trihelix genes showed the tendency for ubiquitous expression in most plant organs, but rarely in pollen (Supplementary Fig. S6A). Furthermore, many rice trihelix genes in Clades I (Os04g45750, Os02g01380, Os02g43300, Os03g02240, and Os10g37240), II (Os04g51320), IV (Os04g32590 and Os02g07800), and V (Os05g48690, Os01g48320, Os09g38570, and Os04g45940) showed broad transcriptional responses against abiotic stresses, such as cold, drought, heat, and salt. Although the responses of individual genes varied according to the particular stressor employed (Supplementary Fig. S6B). Unexpectedly, the expression of *Arabidopsis* genes was rarely stimulated by abiotic stress, even though the transcripts of several genes (AT5G28300 and AT1G33240 in Clade I, AT2G38250 and AT5G01380 in Clade II) in root samples were shown to be up-regulated under drought or salt conditions (Supplementary Fig. S5B). For further validation, we next selected 10 genes in rice and their orthologs in sorghum to conduct semi-quantitative RT–PCR analysis with leaf tissue from seedlings subjected to high salinity (250 mM NaCl), dehydration (25% of PEG 10,000), and cold (4°C) conditions. Results similar to microarray data were obtained for the selected rice trihelix genes, in that all clades demonstrated stress-induced expression changes (Fig. 4). In Clade I, the expression of both Os04g45750 and Os02g43300 were stimulated by cold, salt, and drought conditions, whereas expression in the sorghum homologs, Sb06g023980 and Sb04g033390, were not influenced by any of the stressors. The expression of Os02g33610 was not induced by any of the tested stressors, while its ortholog, Sb04g005900, preserved the expression motif against cold and salt. In Clade II, the expression levels of Os04g40930 were decreased compared with those of the sorghum ortholog, Sb06g020670. In contrast, the orthologous pair of Os04g51320 and Sb06g027540, conserved the expression pattern in response to cold, drought, and salt (Fig. 4). Similar results were also observed in Clades III, IV, and V, where the expression between each gene member in the same clade or between orthologs showed a large divergence (Fig. 4). To obtain additional information regarding the functions of the trihelix family, we conducted co-expression analysis using rice microarray data. As expected, Os01g52090 and Os02g07800 were co-expressed with abiotic and biotic stress-related genes. Several genes of Os02g07800, Os05g48690, and Os02g33610 were found to be co-expressed with genes involved in nitrogen compound metabolic processes. Furthermore, the functional enrichment tests suggested that Os01g21590 and Os01g48320 were associated with the oxidation–reduction processes and the ubiquitin-dependent protein catabolic processes, respectively, while Os09g38570, Os04g45750, and Os10g37240 were implicated in glycosylase activity and microtubule motor activity (Supplementary Table S7).

### 3.4. Functional characterization of transgenic *planta*

**Figure 5.**
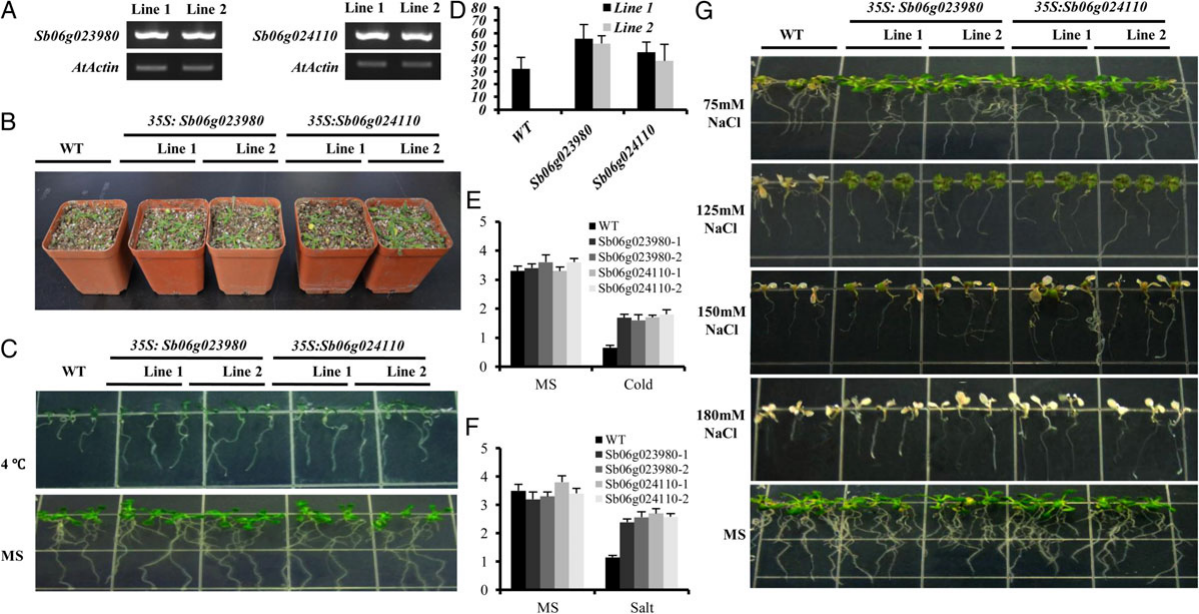
Performance of Sb6g023980 and Sb06g024110 transgenic plants under drought, salt, and cold stresses. (A) Validation of the expression level of Sb6g023980 and Sb06g024110 in transgenic *Arabidopsis*. (B) Phenotype of the transgenic plants under drought condition. (C) Phenotype of the transgenic plants under cold stress (4°C). (D) Survival rate of the transgenic plants under drought stress. Each data point is the average of three experiments and bars indicate SD. (E) Root length of the transgenic plants under cold stress. Each data point is the average of three experiments and bars indicate SD. (F) Root length of the transgenic plants under salt stress. Each data point is the average of three experiments and bars indicate SD. (G) Phenotype of the transgenic plants under salt stress.

The transcriptional responses to stress may be informative about the functions of trihelix genes. Here, both the Sb06g023980 in Clade I and Sb06g024110 in Clade V showed the response against cold, salt, or drought. They harbour one or two GT domains, respectively. To further examine their functions, we generated transgenic *Arabidopsis* overexpressing lines (*35S:Sb06g023980* and *35S:Sb06g024110*) for stress tolerance test. Two-independent transgenic lines (T_3_) were selected depending on the expression levels under normal conditions (Fig. 5A). When grown at normal conditions in soil, no significant difference in phenotypes was observed between wild-type and transgenic plants. However, after exposure to drought stress (i.e. stopping irrigation for 10 days), the transgenic plants exhibited a higher tolerance than control plants (Fig. 5B). After watering again, the survival rates of Sb06g023980 transgenic plants reached 55.7% (34/61 for Line 1) and 51.9% (30/57 for Line 2), while Sb06g024110 transgenic plants reached 45.1% (23/51 for Line 1) and 38.2% (24/63 for Line 2), both of which were significantly higher than those of the control plants (32.1%, 27/84), respectively (Fig. 5D). For cold and salt tolerance, the root length test showed that the transgenic seedlings grew faster over a 7-day period and their roots were significantly longer (*t*-test, *P* < 0.01) than the wild type. No significant difference was observed between the transgenic and the wild-type seedlings under normal conditions (Fig. 5E–G). These results imply that heterogeneous overexpression of Sb06g023980 and Sb06g024110 genes in *Arabidopsis* can promote the rates of seedling growth and survival under drought, cold, and salt stress conditions.

## Discussion

### Trihelix family in *planta*

Generally, trihelix TFs are thought to feature a typical DNA-binding structure of trihelix (helix-loop-helix-loop-helix) domain;^3^ however, they are classified together with myb/SANT-like domains in Pfam (PF13837). By taking advantage of the recently sequenced plant genomes, we hereby identified the family members on the basis of sequence homology using the putative trihelix genes as a blast query. A total of 319 genes were found covering all of the tested land plants, while no algae trihelix genes were discovered. This result implies that the trihelix genes may have emerged since land plants during evolution. Interestingly, only eight genes in the EST database were identified for wheat, which is a hexaploid and should contain 2-3 times the number of gene members than diploid grass species, such as rice (30) and sorghum (28). One reason for this discrepancy may be incomplete profiling of the wheat gene, although the EST database reportedly covers ∼95% of wheat transcriptome.^43^

The trihelix family had previously been classified, using rice and *Arabidopsis* genes, into three distinctive subfamilies (GTα, GTβ, and GTγ).^27^ Here, we employed all the tested species in the analyses, covering the trihelix genes from moss, fern, and monocot and dicot plants. After applying the BIC and AIC test and constructing a phylogenetic tree using the ML procedure to classify the genes into five major clades, we used the JTT model of amino acid substitutions to evaluate phylogeny (Fig. 1). Upon examining the *Arabidopsis* and rice genes, we found that Clades II, IV, and V harbour the genes in α-subfamily, while Clades I and III mainly correspond to the β and γ subfamily. Because all the Clades I, IV, and V contain the trihelix genes in ancestral species at rooting positions, and thus we prefer to classify them in three different clades (Fig. 1). Similar to our study, Kaplan-levy *et al.* classified trihelix genes from rice and *Arabidopsis* into five clades, named GT-2, GT-1, SH4, SIP1, and GTγ. However, that classification method placed the sub-branch containing At5g63420 and Os02g33610 (Clade I in the current study) into the GT-1 clade (Clade II in the current study).^11^ Our analysis revealed that two additional sub-branches, located on more rooted position of the aforementioned sub-branch, contain trihelix members only from ancestral species, such as Pp1s29_84V6, 409669 and Pp1s85_150V6. This finding supports the classification of this branch into Clade I (Supplementary Table S2). Furthermore, most of the genes containing a C-terminal trihelix domain were found to fall in Clade I (Supplementary Table S2), additional evidence that this sub-branch is appropriately classified in Clade I.

A total of 31 rice trihelix members had been identified before by Kaplan-levy *et al.* However, we found that Os4g33300, a chimeric gene with a kinase domain at C-terminal, had lost the trihelix domain and was therefore excluded from this family in this study (data not shown).^11^ Moreover, previous reports indicate that there are two types of trihelix genes which harbour one or two GT domains, while the evolutionary turnover of these domains are unclear.^27^ In our study, both the single and double domain structures have been found in ancestral species in Clade I, revealing their early divergence during evolution (Supplementary Table S2).

### Characterization of trihelix genes

The *Poaceae* dates back to about 50–70 million years, with a gene expansion by the pre-grass whole genome duplication in the last common ancestor.^44^ The examination of the duplication blocks of the trihelix family among *O. sativa*, *S. bicolor*, *B. distachyon*, and *Z. mays* revealed that most trihelix gene copies were well preserved in the genome, suggesting the functional conservation of these genes. This conservation is apparent even in the trihelix family in *B. distachyon*, which experienced extensive independent genome reduction during evolution.^45^ In the case of *Z. mays*, a total of 43 collinear genes were detected, including only 13 gene pairs located on homeologous chromosomes. The true functional significance of the partial gene loss between duplication regions in *Z. mays* is unknown, but it is generally thought to be involved in returning an ancient allotetraploid to a genetically diploid state.^41^

In Pfam, the trihelix transcription factor is classified into the myb/SANT-like family, as they all form an α-helix-turn-α-helix structure. However, the trihelix family is generally thought to feature a typical helix-loop-helix-loop-helix structure with an individual helix longer than the myb repeat, which targets different binding sequences.^42^ In our study, the trihelix family was classified into five major clades, all of which showed the typical features of the trihelix family as the previously described, such as the high conservation of tryptophan (W) and an additional F and L rich α-helix lying closely downstream to the trihelix domain (Supplementary Fig. S2). A recently reported solution structure of *Arabidopsis* GT-1 revealed that the three helices were held together by a hydrophobic core, and the third helix was likely responsible for DNA binding with the aid of the fourth helix.^42^ In our structural analysis, we found the predicted 3D structures were relatively conserved (Supplementary Fig. S4), although the sequence of α-helixes and the length of linker sequence between each α-helix were dynamic among clades (Supplementary Fig. S2). When testing the subcellular localization of the selected sorghum trihelix genes from each clade, the strong nuclear signals supported their functional roles in transcriptional regulation. However, the conservation or divergence of the binding sequence of the family members in each clade needs to be further investigated.

### Transcriptional responses of trihelix genes against abiotic stresses

The *Arabidopsis* and rice are model dicot and monocot plants. According to our analysis of the public rice microarray data, Clade I of *Arabidopsis* trihelix genes shows enriched expression in inflorescence, while genes in Clades II–V seemed more abundantly expressed in seeds (Supplementary Fig. S5). This is consistent with previously described functional roles of trihelix genes in flower and seed development.^11,17,18^ Conversely, the expression pattern of rice trihelix genes differs from that in *Arabidopsis* (Supplementary Fig. S6). Most family members in rice were broadly expressed during the plant life cycle and showed an extensive response to abiotic stresses. These findings were further confirmed by RT–PCR (Fig. 4). A robust response to cold, drought, and salt was also detected in gene members of sorghum, although the same expression pattern was not always conserved between sorghum and rice orthologs. It is yet to be determined whether the responses to abiotic stress are the major differences between dicots and monocots of this gene family. There is valuable knowledge to be gained by studying the evolutionary turnover of these functional roles involved in plant developmental programmes and abiotic stress resistance. The transcriptional divergence among the gene members in rice and sorghum is likely due to regulatory subfunctionalization, as it is very unlikely these genes independently evolved all of the functional roles in cold, drought, and salt tolerance.

### Functional characterization of transgenic *planta*

Recently, several trihelix genes from rice (in Clade III) and soybean (in Clade I) were reported to be involved in salt and drought tolerance,^21,22^ which broadens the functional roles of this family. Our transcriptional analysis on rice and sorghum revealed that the expression of trihelix genes within various clades was broadly stimulated by abiotic stress (Fig. 4), which implies that the functional involvement in abiotic stress resistance within this family might be conserved in grass. This supposition was further supported by the phenotypic analysis of transgenic *Arabidopsis* overexpressing Sb06g024110 (one GT domain) in Clade V and Sb06g023980 (two GT domains) in Clade I. Both demonstrated increased drought, salt, and cold tolerance (Fig. 5). In contrast, our transcriptional study of Sb06g023980 showed that gene expression was not stimulated by drought (Fig. 4). This further supports the regulatory subfunctionalization of this gene family in sorghum.

In this study, we established the trihelix family radiation in 10 plant species and carried out functional characterization. The current trihelix family, expanded in angiosperm, seems to be a common ancestor of land plants with a large functional divergence associated with regulation of light, stress, and a series of developmental programmes.^11^ The results of our current study indicate functional conservation within this family in transcriptional regulation responses to drought, salt, and cold. Furthermore, we believe this conservation to be of ancestral origin, rather than a newly evolved phenomenon. The extent of the functional diversification within the trihelix family across different species is a topic for further investigation. Results of our co-expression analysis suggest that examining the involvement of this gene family in nitrogen metabolic processes, oxidation–reduction processes, and glycosylase and microtubule motor activity may add to the existing evolutionary, transcriptional, and functional body of knowledge surrounding the trihelix transcription factor family.

## Supplementary data

Supplementary data are available at www.dnaresearch.oxfordjournals.org

## Funding

This work was financially supported by the National Basic Research Program of China (973 Program, 2014CB138100) and the National Natural Science Foundation of China (no. 31200982). Also, thanks to the Korea University grant supported to W.K.

## Supplementary Material

Supplementary Data
