# Supplementary material for: Evolutionary History of Trihelix Family and Their Functional Diversification
Source: DNA Res. 2014 May 25;21(5):499–510. doi: 10.1093/dnares/dsu016 (PMC4195496; doi:10.1093/dnares/dsu016)
Supplement: Supplementary Data [file supp_21_5_499__index.html]

Evolutionary History of Trihelix Family and Their Functional Diversification — Evolutionary History of Trihelix Family and Their Functional Diversification — Supplementary Data 

# Evolutionary History of Trihelix Family and Their Functional Diversification

## Supplementary Data

Supplementary Data

**Files in this Data Supplement:**

- Supplementary Figures - ppt file
- Supplementary Table 1 - doc file
- Supplementary Table 2-4 - doc file
- Supplementary Table 5-6 - doc file
- Supplementary Table 7 - xls file
