# Supplementary material for: Evolutionary History of Trihelix Family and Their Functional Diversification
Source: DNA Res. 2014 May 25;21(5):499–510. doi: 10.1093/dnares/dsu016 (PMC4195496; doi:10.1093/dnares/dsu016)
Supplement: Supplementary Data [file supp_dsu016_dsu016supp_figs.ppt]

## Slide 1
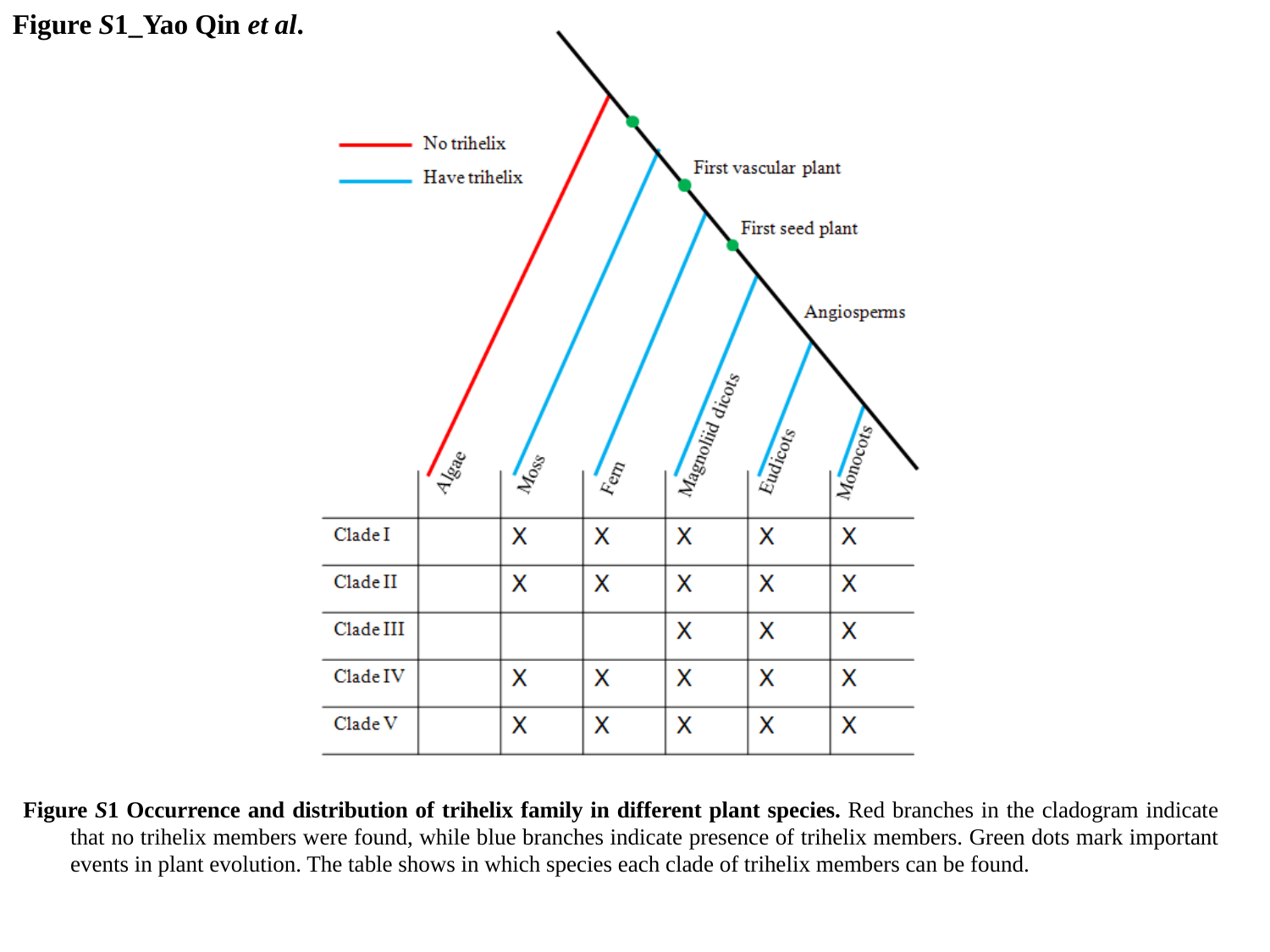

Figure S1_Yao Qin et al.
Figure S1 Occurrence and distribution of trihelix family in different plant species. Red branches in the cladogram indicate that no trihelix members were found, while blue branches indicate presence of trihelix members. Green dots mark important events in plant evolution. The table shows in which species each clade of trihelix members can be found.

## Slide 2
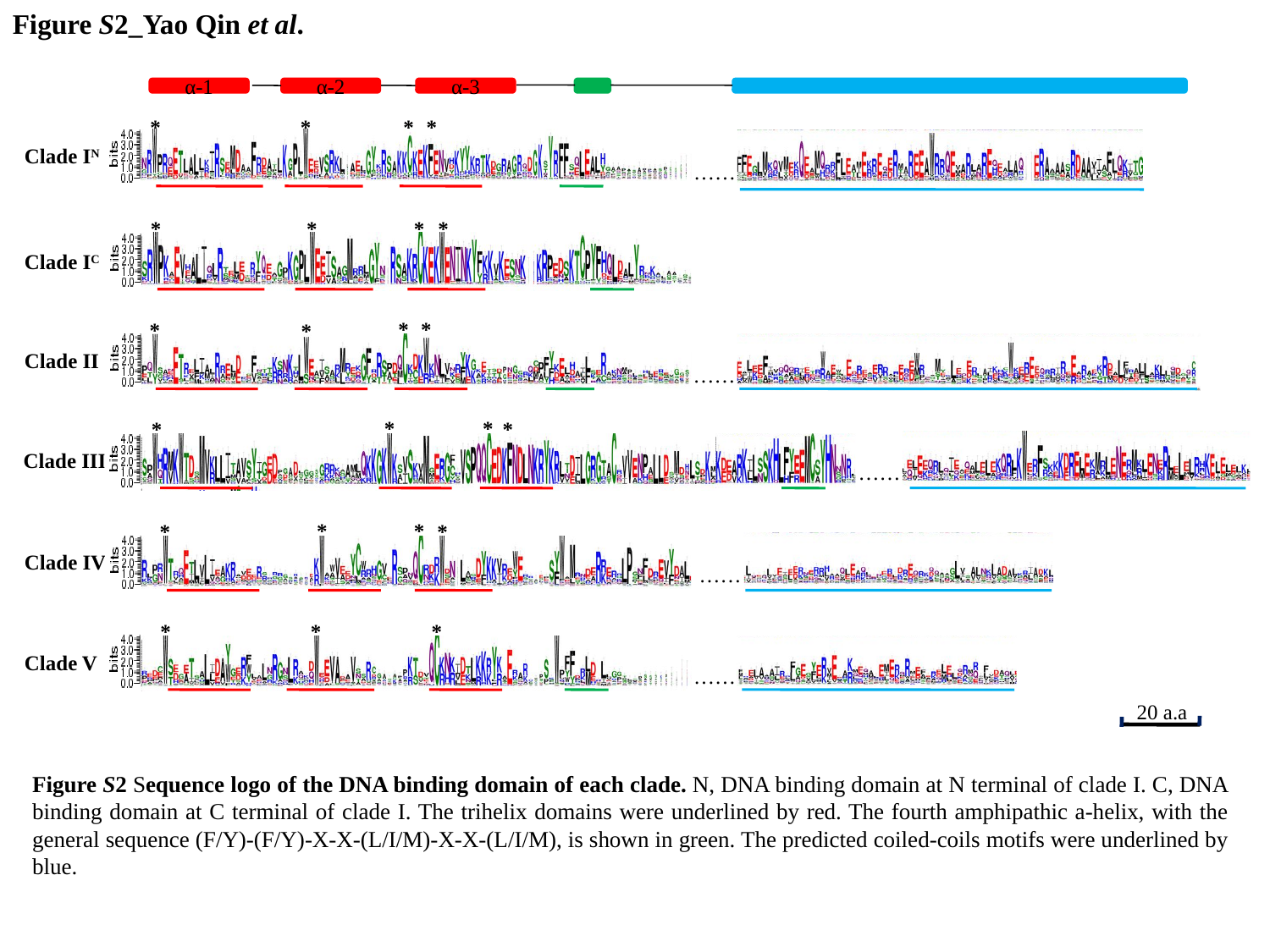

Figure S2_Yao Qin et al.
α-1
α-2
α-3
*
*
*
*
Clade IN
……
*
*
*
*
Clade IC
*
*
*
*
Clade II
……
*
*
*
*
Clade III
……
*
*
*
*
Clade IV
……
*
*
*
Clade V
……
20 a.a
Figure S2 Sequence logo of the DNA binding domain of each clade. N, DNA binding domain at N terminal of clade I. C, DNA binding domain at C terminal of clade I. The trihelix domains were underlined by red. The fourth amphipathic a-helix, with the general sequence (F/Y)-(F/Y)-X-X-(L/I/M)-X-X-(L/I/M), is shown in green. The predicted coiled-coils motifs were underlined by blue.

## Slide 3
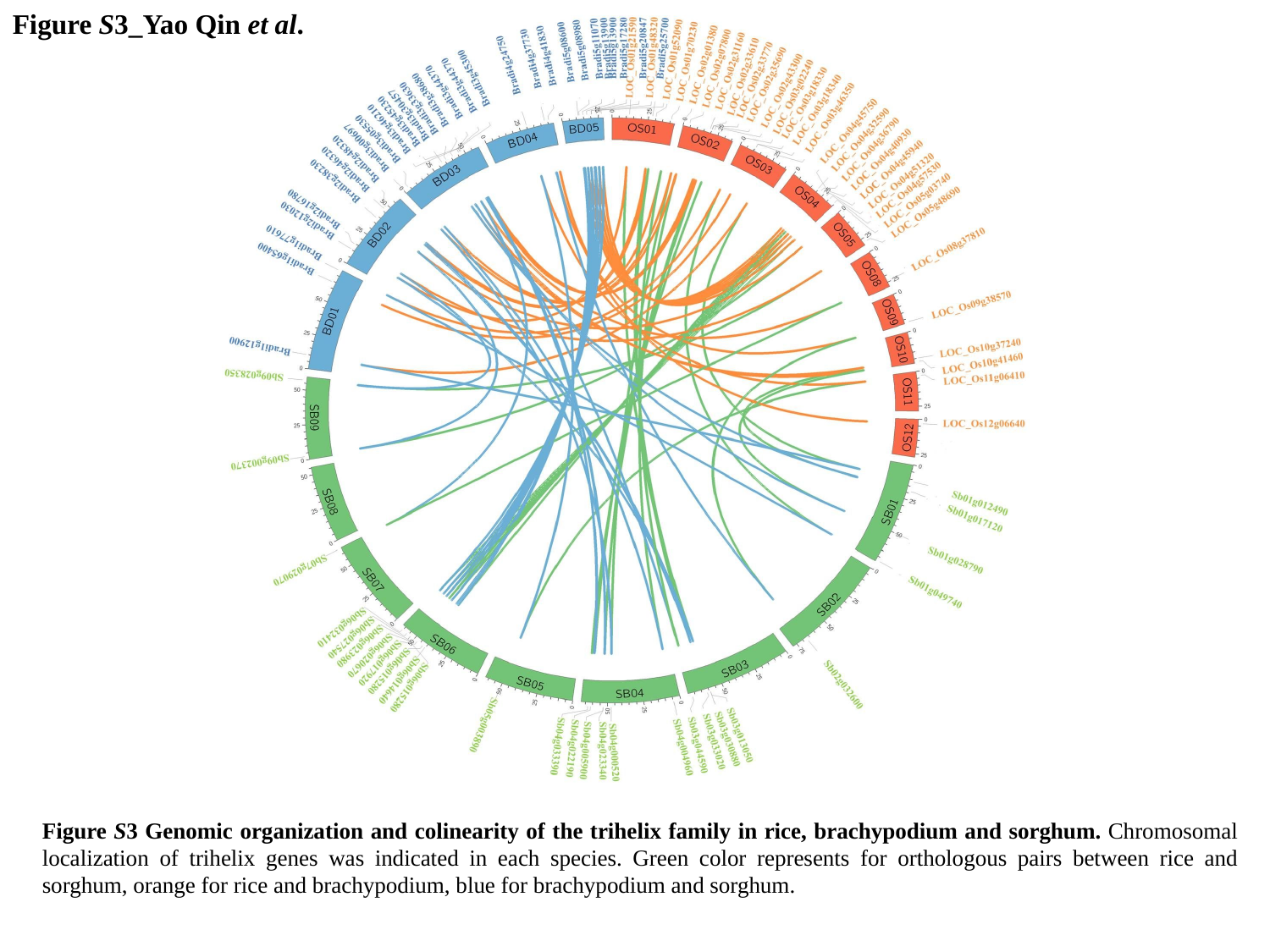

Figure S3_Yao Qin et al.
Figure S3 Genomic organization and colinearity of the trihelix family in rice, brachypodium and sorghum. Chromosomal localization of trihelix genes was indicated in each species. Green color represents for orthologous pairs between rice and sorghum, orange for rice and brachypodium, blue for brachypodium and sorghum.

## Slide 4
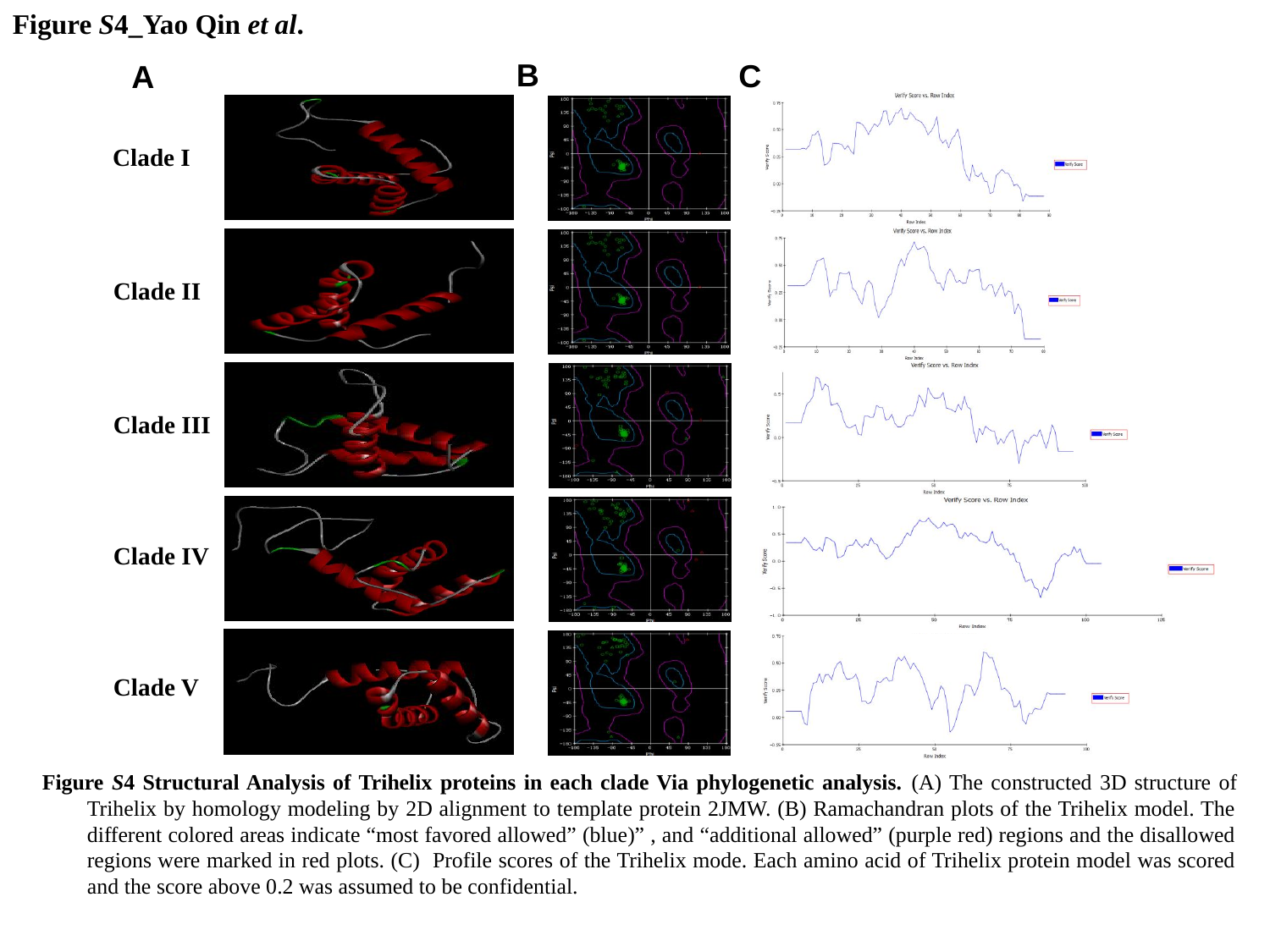

Figure S4_Yao Qin et al.
B
C
A
Clade I
Clade II
Clade III
Clade IV
Clade V
Figure S4 Structural Analysis of Trihelix proteins in each clade Via phylogenetic analysis. (A) The constructed 3D structure of Trihelix by homology modeling by 2D alignment to template protein 2JMW. (B) Ramachandran plots of the Trihelix model. The different colored areas indicate “most favored allowed” (blue)” , and “additional allowed” (purple red) regions and the disallowed regions were marked in red plots. (C) Profile scores of the Trihelix mode. Each amino acid of Trihelix protein model was scored and the score above 0.2 was assumed to be confidential.

## Slide 5
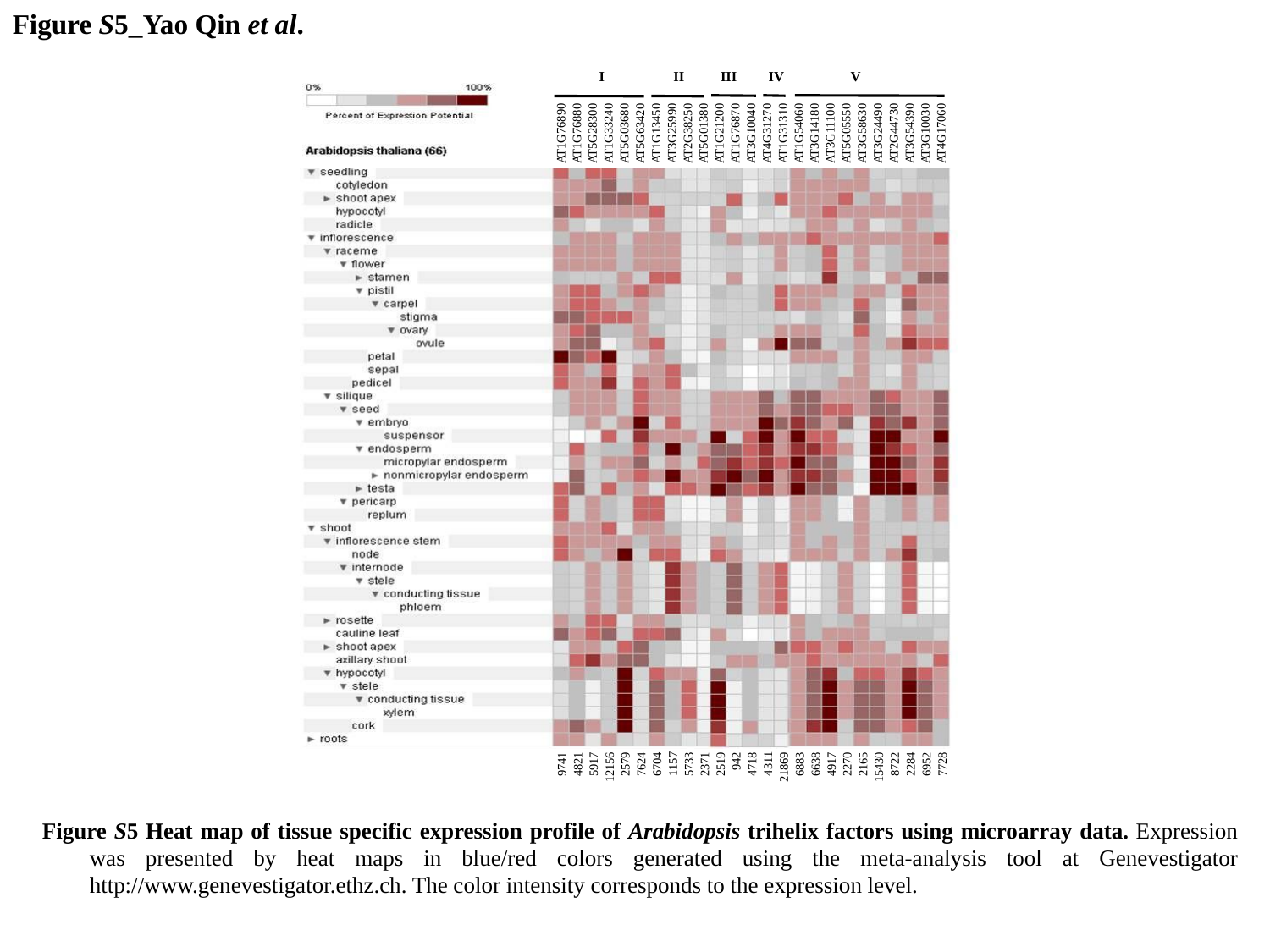

Figure S5_Yao Qin et al.
AT1G76890
AT1G76880
AT5G28300
AT1G33240
AT5G03680
AT5G63420
AT1G13450
AT3G25990
AT2G38250
AT5G01380
AT1G21200
AT1G76870
AT3G10040
AT4G31270
AT1G31310
AT1G54060
AT3G14180
AT3G11100
AT5G05550
AT3G58630
AT3G24490
AT2G44730
AT3G54390
AT3G10030
AT4G17060
I
II
V
III
IV
9741
4821
5917
12156
2579
7624
6704
1157
5733
2371
2519
942
4718
4311
21869
6883
6638
4917
2270
2165
15430
8722
2284
6952
7728
Figure S5 Heat map of tissue specific expression profile of Arabidopsis trihelix factors using microarray data. Expression was presented by heat maps in blue/red colors generated using the meta-analysis tool at Genevestigator http://www.genevestigator.ethz.ch. The color intensity corresponds to the expression level.

## Slide 6
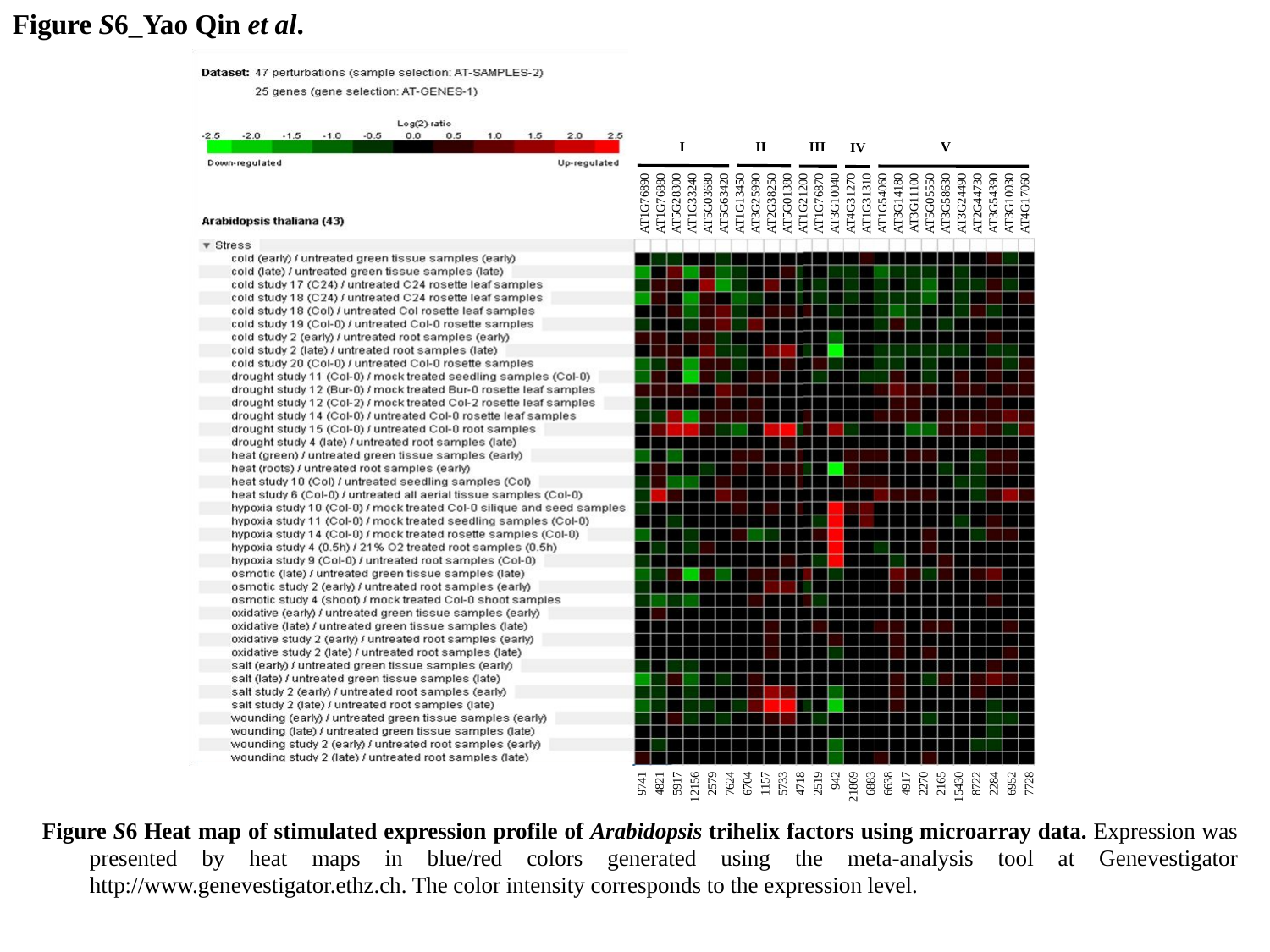

Figure S6_Yao Qin et al.
AT1G76890
AT1G76880
AT5G28300
AT1G33240
AT5G03680
AT5G63420
AT1G13450
AT3G25990
AT2G38250
AT5G01380
AT1G21200
AT1G76870
AT3G10040
AT4G31270
AT1G31310
AT1G54060
AT3G14180
AT3G11100
AT5G05550
AT3G58630
AT3G24490
AT2G44730
AT3G54390
AT3G10030
AT4G17060
I
II
V
III
IV
9741
4821
5917
12156
2579
7624
6704
1157
5733
4718
2519
942
21869
6883
6638
4917
2270
2165
15430
8722
2284
6952
7728
Figure S6 Heat map of stimulated expression profile of Arabidopsis trihelix factors using microarray data. Expression was presented by heat maps in blue/red colors generated using the meta-analysis tool at Genevestigator http://www.genevestigator.ethz.ch. The color intensity corresponds to the expression level.

## Slide 7
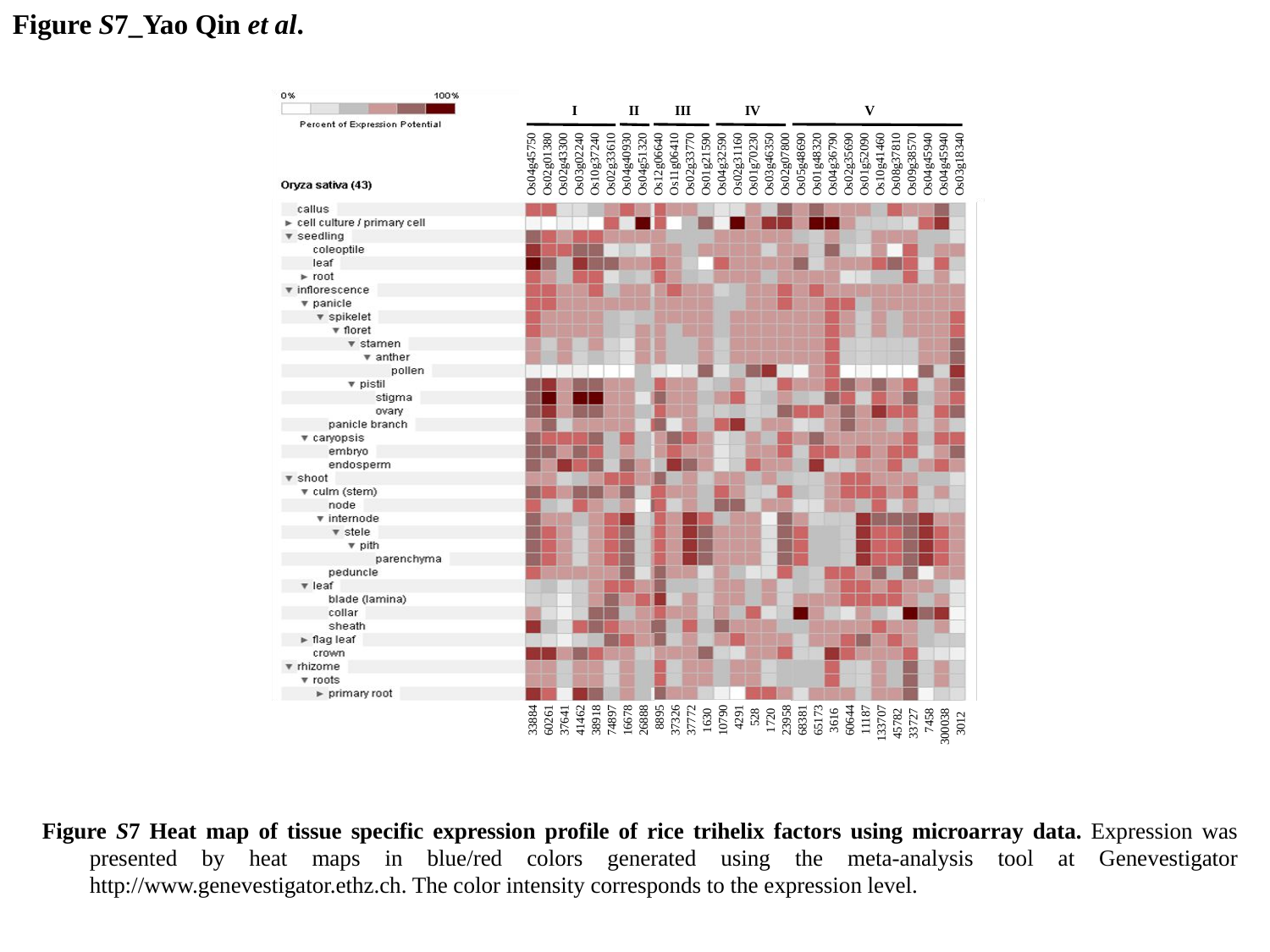

Figure S7_Yao Qin et al.
Os04g45750
Os02g01380
Os02g43300
Os03g02240
Os10g37240
Os02g33610
Os04g40930
Os04g51320
Os12g06640
Os11g06410
Os02g33770
Os01g21590
Os04g32590
Os02g31160
Os01g70230
Os03g46350
Os02g07800
Os05g48690
Os01g48320
Os04g36790
Os02g35690
Os01g52090
Os10g41460
Os08g37810
Os09g38570
Os04g45940
Os04g45940
Os03g18340
I
II
III
IV
V
33884
60261
37641
41462
38918
74897
16678
26888
8895
37326
37772
1630
10790
4291
528
1720
23958
68381
65173
3616
60644
11187
133707
45782
33727
7458
300038
3012
Figure S7 Heat map of tissue specific expression profile of rice trihelix factors using microarray data. Expression was presented by heat maps in blue/red colors generated using the meta-analysis tool at Genevestigator http://www.genevestigator.ethz.ch. The color intensity corresponds to the expression level.

## Slide 8
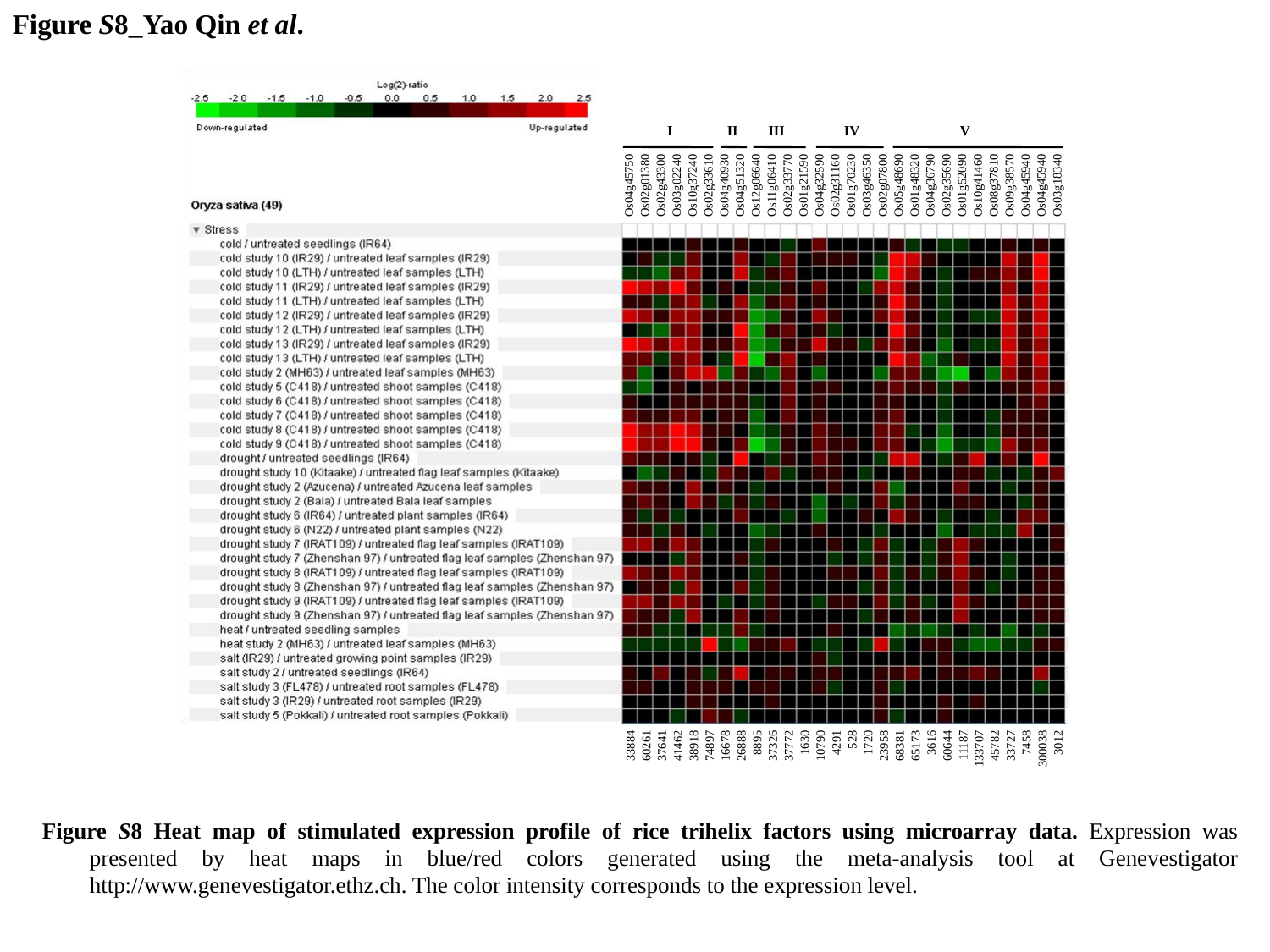

Figure S8_Yao Qin et al.
Os04g45750
Os02g01380
Os02g43300
Os03g02240
Os10g37240
Os02g33610
Os04g40930
Os04g51320
Os12g06640
Os11g06410
Os02g33770
Os01g21590
Os04g32590
Os02g31160
Os01g70230
Os03g46350
Os02g07800
Os05g48690
Os01g48320
Os04g36790
Os02g35690
Os01g52090
Os10g41460
Os08g37810
Os09g38570
Os04g45940
Os04g45940
Os03g18340
III
I
II
IV
V
33884
60261
37641
41462
38918
74897
16678
26888
8895
37326
37772
1630
10790
4291
528
1720
23958
68381
65173
3616
60644
11187
133707
45782
33727
7458
300038
3012
Figure S8 Heat map of stimulated expression profile of rice trihelix factors using microarray data. Expression was presented by heat maps in blue/red colors generated using the meta-analysis tool at Genevestigator http://www.genevestigator.ethz.ch. The color intensity corresponds to the expression level.
