# Supplementary material for: Evolutionary History of Trihelix Family and Their Functional Diversification
Source: DNA Res. 2014 May 25;21(5):499–510. doi: 10.1093/dnares/dsu016 (PMC4195496; doi:10.1093/dnares/dsu016)
Supplement: Supplementary Data [file supp_dsu016_dsu016supp_table1.doc]

**Supplementary Table 1 Primers for amplification of trihelix gene fragments in RT-PCR**

| **Group** | **Gene ID** | **RT-PCR primers** | **RT-PCR Condition** |
| --- | --- | --- | --- |
| **I** | *Os04g45750* | CGCGGGACAACAAGAAGAAC | 27 cycle/57℃ for annealing |
|  |  | CTTTGTCCTTGGACGCCTTG |  |
|  | *Sb06g023980* | AGTCGAAGCCAATCTCAGCC | 27 cycle /58℃ for annealing |
|  |  | GAGCTCCGGTTGTATCCCAG |  |
|  | *Os02g43300* | CGCGGGACAACAAGAAGAAC | 27 cycle /57℃ for annealing |
|  |  | CTTTGTCCTTGGACGCCTTG |  |
|  | *Sb04g033390* | TCGGACGACTACGACTACGA | 30 cycle /57℃ for annealing |
|  |  | CTCGTTCCGCATCTGGATCA |  |
|  | *Os02g33610* | CGCGGGACAACAAGAAGAAC | 30 cycle /57℃ for annealing |
|  |  | CTTTGTCCTTGGACGCCTTG |  |
|  | *Sb04g005900* | AACCCCTCCTAAGGCAGTGA | 29 cycle /58℃ for annealing |
|  |  | CCCCCATTGACAATTCGTGC |  |
| **II** | *Os04g40930* | CGCGGGACAACAAGAAGAAC | 29 cycle /57℃ for annealing |
|  |  | CTTTGTCCTTGGACGCCTTG |  |
|  | *Sb06g020670* | AGTCCAACAAGCACCTCTGG | 28 cycle /57℃ for annealing |
|  |  | ATTGGATGTGGGGCTCTTCC |  |
|  | *Os04g51320* | CGCGGGACAACAAGAAGAAC | 29 cycle /57℃ for annealing |
|  |  | CTTTGTCCTTGGACGCCTTG |  |
|  | *Sb06g027540* | GTGCAGCAACAGCACATTGA | 28 cycle /58℃ for annealing |
|  |  | CTTTGCTCCTCCCGTTCCAT |  |
| **III** | *Os02g33770* | CGCGGGACAACAAGAAGAAC | 30 cycle /57℃ for annealing |
|  |  | CTTTGTCCTTGGACGCCTTG |  |
|  | *Sb04g022190* | CCACTTCGATCTGGGGCAG | 28 cycle /57℃ for annealing |
|  |  | CTCGTCGATGTAGGACACGG |  |
| **IV** | *Os02g07800* | GAGATTGGTGAAAAGAGCGCC | 30 cycle /57℃ for annealing |
|  |  | TCCCGTTTCGCAGACTTCAT |  |
|  | *Sb04g004960* | GAGGCCGAGGTTGGAAAAGA | 29 cycle /57℃ for annealing |
|  |  | CCATTGCATCAGGCAGCATC |  |
| **V** | *Os05g48690* | TGATGCTGAGGAGATGTTGAGATT | 26 cycle /60℃ for annealing |
|  |  | AACGCAACATCAGGGGCTTA |  |
|  | *Sb09g028350* | TCCTCAATTCGCTTTGCCCT | 27 cycle /60℃ for annealing |
|  |  | CTCCACGCGCTCATACATCT |  |
|  | *Os01g48320* | GAGATGTACGAGCGCATGGA | 28 cycle /56℃ for annealing |
|  |  | CATCCGCTTCAGCTCCAGAT |  |
|  | *Sb03g030880* | TGGAGAGGCAGCGAATCAAG | 27v/58℃ for annealing |
|  |  | CAGTGCAGCGAGAGACATGA |  |
|  | *Os04g45940* | GAGTAAATCTCGGTGGCGCA | 35 cycle /58℃ for annealing |
|  |  | TACGATTTCCAAGCTACGCCT |  |
|  | *Sb06g024110* | ATGAGGAAGGTGTGAGCGAC | 29 cycle /58℃ for annealing |
|  |  | TGAGGAGACACCTTTGTGCC |  |
| **House Keeping** | *OSATN* | TCCATCTTGGCATCTCTCAG | 28 cycle /60℃ for annealing |
|  |  | GTACCCTCATCAGGCATCTG |  |
|  | *SbATN* | TGATGAAGATTCTCACTGAG | 28 cycle /60℃ for annealing |
|  |  | GATCCACATCTGTTGGAACG |  |
