# Supplementary material for: Evolutionary History of Trihelix Family and Their Functional Diversification
Source: DNA Res. 2014 May 25;21(5):499–510. doi: 10.1093/dnares/dsu016 (PMC4195496; doi:10.1093/dnares/dsu016)
Supplement: Supplementary Data [file supp_dsu016_dsu016supp_table2-4.doc]

**Supplementary Table 2** Unrooted phylogenetic tree of trihelix gene members of clade I.

| **Gene ID** | **Species** | **Name** | **Length** | **Mass** | **PI** | **1 200 400 600 (a.a)** |
| --- | --- | --- | --- | --- | --- | --- |
|  | *S. bicolor* | *SbTri01* | 673 | 70.71 | 9.39 | 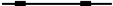 |
|  | *Z. maize* | *GrTri01* | 725 | 76.40 | 6.73 | 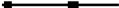 |
|  | *S. italica* | *SiTri01* | 753 | 96.36 | 8.79 | 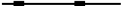 |
|  | *O. sativa* | *OsTri01* | 725 | 76.49 | 6.63 | 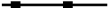 |
|  | *T. aestivum* | *TaTri01* | 797 | 85.22 | 7.15 | 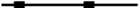 |
|  | *B. distachyon* | *BdTri01* | 635 | 67.29 | 9.83 | 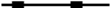 |
|  | *A. caerulea* | *AcTri01* | 796 | 87.26 | 5.82 | *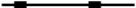* |
|  | *B. distachyon* | *BdTri02* | 769 | 82.09 | 5.72 | 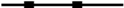 |
|  | *T. aestivum* | *TaTri02* | 792 | 84.17 | 6.14 | 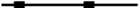 |
|  | *S. bicolor* | *SbTri02* | 807 | 85.11 | 6.23 | 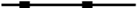 |
|  | *Z. maize* | *GrTri02* | 777 | 82.58 | 6.11 | 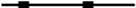 |
|  | *A. thaliana* | *AtTri01* | 669 | 74.21 | 5.73 | 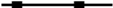 |
|  | *S. bicolor* | *SbTri03* | 770 | 82.63 | 5.85 | 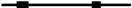 |
|  | *Z. maize* | *GrTri03* | 774 | 83.24 | 5.90 | 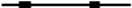 |
|  | *B. distachyon* | *BdTri03* | 758 | 81.89 | 6.11 | 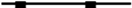 |
|  | *S. italica* | *SiTri02* | 675 | 72.18 | 5.99 | 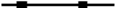 |
|  | *O. sativa* | *OsTri02* | 628 | 67.74 | 4.87 | 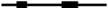 |
|  | *S. italica* | *SiTri03* | 665 | 71.65 | 5.58 | 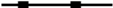 |
|  | *S. italica* | *SiTri04* | 590 | 68.24 | 5.67 | 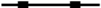 |
|  | *Z. maize* | *GrTri04* | 668 | 71.71 | 5.78 | 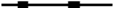 |
|  | *S. bicolor* | *SbTri04* | 720 | 76.75 | 5.59 | 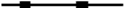 |
|  | *Z. maize* | *GrTri05* | 672 | 72.81 | 5.87 | 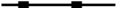 |
|  | *P. patens* | *PpTri01* | 742 | 82.22 | 8.15 | 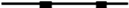 |
|  | *P. patens* | *PpTri02* | 757 | 83.76 | 7.48 | 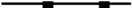 |
|  | *O. sativa* | *OsTri03* | 860 | 88.76 | 5.57 | 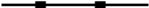 |
|  | *M. guttatus* | *MgTri01* | 506 | 57.13 | 6.55 | 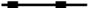 |
|  | *A. thaliana* | *AtTri02* | 575 | 65.84 | 6.35 | 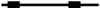 |
|  | *A. thaliana* | *AtTri03* | 603 | 67.88 | 6.30 | 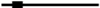 |
|  | *M. guttatus* | *MgTri02* | 604 | 66.84 | 6.35 | 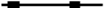 |
|  | *M. guttatus* | *MgTri03* | 656 | 72.08 | 6.14 | 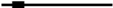 |
|  | *A. caerulea* | *AcTri02* | 535 | 60.20 | 5.41 | 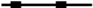 |
|  | *S. moellendorffii* | *SmTri01* | 324 | 34.94 | 9.29 | 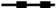 |
|  | *S. moellendorffii* | *SmTri02* | 216 | 25.12 | 9.78 | 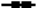 |
|  | *A. thaliana* | *AtTri04* | 619 | 71.28 | 6.74 | 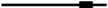 |
|  | *M. guttatus* | *MgTri04* | 546 | 62.25 | 6.37 | 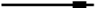 |
|  | *A. caerulea* | *AcTri03* | 597 | 68.41 | 5.88 | 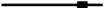 |
|  | *S. italica* | *SiTri05* | 246 | 28.24 | 9.62 | 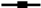 |
|  | *O. sativa* | *OsTri04* | 323 | 38.27 | 6.11 | 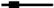 |
|  | *B. distachyon* | *BdTri04* | 758 | 80.53 | 5.88 | 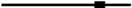 |
|  | *Z. maize* | *GrTri06* | 714 | 76.27 | 5.93 | 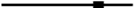 |
|  | *Z. maize* | *GrTri07* | 664 | 70.65 | 5.67 | 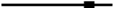 |
|  | *S. bicolor* | *SbTri05* | 740 | 78.47 | 5.88 | 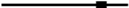 |
|  | *S. moellendorffii* | *SmTri03* | 213 | 25.58 | 9.50 | 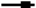 |
|  | *O. sativa* | *OsTri05* | 528 | 57.46 | 5.74 | 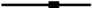 |
|  | *A. thaliana* | *AtTri05* | 398 | 45.58 | 5.91 | 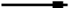 |
|  | *A. caerulea* | *AcTri04* | 591 | 66.87 | 5.95 | 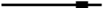 |
|  | *A. caerulea* | *AcTri05* | 510 | 58.18 | 5.78 | 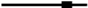 |
|  | *A. thaliana* | *AtTri06* | 481 | 55.50 | 7.69 | 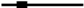 |
|  | *A. thaliana* | *AtTri07* | 591 | 66.64 | 6.63 | 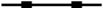 |
|  | *M. guttatus* | *MgTri05* | 539 | 61.33 | 8.37 | 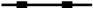 |
|  | *M. guttatus* | *MgTri06* | 532 | 59.92 | 6.42 | 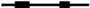 |
|  | *S. moellendorffii* | *SmTri04* | 829 | 93.49 | 8.91 | 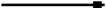 |
|  | *A. thaliana* | *AtTri08* | 911 | 100.55 | 8.42 | 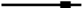 |
|  | *M. guttatus* | *MgTri07* | 869 | 96.16 | 8.32 | 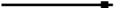 |
|  | *A. caerulea* | *AcTri06* | 969 | 107.55 | 6.51 | 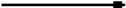 |
|  | *T. aestivum* | *TaTri03* | 394 | 44.43 | 8.88 | 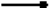 |
|  | *B. distachyon* | *BdTri05* | 875 | 96.63 | 8.90 | 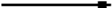 |
|  | *O. sativa* | *OsTri06* | 882 | 97.37 | 8.97 | 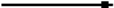 |
|  | *S. italica* | *SiTri06* | 878 | 96.36 | 8.79 | 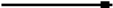 |
|  | *Z. maize* | *GrTri08* | 510 | 56.49 | 8.81 | 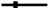 |
|  | *S. bicolor* | *SbTri06* | 875 | 96.29 | 9.04 | 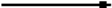 |
|  | *Z. maize* | *GrTri09* | 695 | 76.87 | 9.04 | 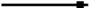 |
|  | *S. moellendorffii* | *SmTri05* | 552 | 60.93 | 9.30 | 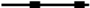 |
|  | *P. patens* | *PpTri03* | 496 | 56.55 | 8.83 | 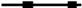 |
|  | *P. patens* | *PpTri04* | 514 | 58.54 | 8.67 | 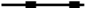 |
|  | *P. patens* | *PpTri05* | 498 | 56.21 | 6.77 | 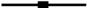 |
|  | *P. patens* | *PpTri06* | 499 | 56.01 | 6.55 | 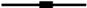 |
|  |  |  |  |  |  |  |

**Supplementary Table 3 Unrooted phylogenetic tree of trihelix gene members of clade II.**

| **Gene ID** | **Species** | **Name** | **Length** | **Mass** | **PI** | **1 200 400 600 (a.a)** |
| --- | --- | --- | --- | --- | --- | --- |
|  | *S. bicolor* | *SbTri07* | 271 | 31.40 | 7.73 | *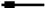* |
|  | *Z. maize* | *GrTri10* | 273 | 32.31 | 8.37 | *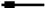* |
|  | *Z. maize* | *GrTri11* | 271 | 31.90 | 8.94 | *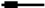* |
|  | *S. italica* | *SiTri07* | 273 | 31.85 | 7.73 | *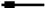* |
|  | *S. italica* | *SiTri08* | 247 | 31.84 | 7.72 | *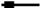* |
|  | *O. sativa* | *OsTri07* | 277 | 32.27 | 6.95 | *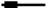* |
|  | *B. distachyon* | *BdTri06* | 275 | 32.09 | 8.97 | *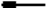* |
|  | *M. guttatus* | *MgTri08* | 270 | 32.58 | 9.53 | *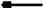* |
|  | *P. patens* | *PpTri07* | 310 | 37.18 | 7.67 | *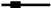* |
|  | *S. moellendorffii* | *SmTri11* | 307 | 36.29 | 5.88 | *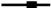* |
|  | *S. moellendorffii* | *SmTri12* | 297 | 35.43 | 6.84 | *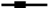* |
|  | *P. patens* | *PpTri08* | 344 | 40.92 | 5.73 | *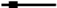* |
|  | *P. patens* | *PpTri09* | 325 | 38.71 | 5.99 | *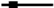* |
|  | *P. patens* | *PpTri10* | 289 | 34.98 | 6.96 | *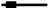* |
|  | *P. patens* | *PpTri11* | 305 | 36.65 | 5.82 | *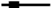* |
|  | *M. guttatus* | *MgTri09* | 293 | 34.29 | 7.75 | *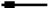* |
|  | *M. guttatus* | *MgTri10* | 268 | 31.56 | 8.50 | *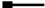* |
|  | *A. thaliana* | *AtTri09* | 323 | 38.27 | 6.11 | *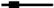* |
|  | *A. thaliana* | *AtTri10* | 289 | 34.31 | 6.55 | *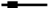* |
|  | *O. sativa* | *OsTri08* | 333 | 36.95 | 5.85 | *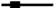* |
|  | *Z. maize* | *GrTri12* | 673 | 76.47 | 9.57 | *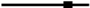* |
|  | *T. aestivum* | *TaTri04* | 322 | 36.01 | 5.77 | *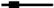* |
|  | *B. distachyon* | *BdTri07* | 345 | 38.46 | 6.07 | *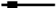* |
|  | *S. italica* | *SiTri09* | 412 | 46.36 | 10.09 | *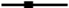* |
|  | *Z. maize* | *GrTri13* | 335 | 36.76 | 5.95 | *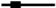* |
|  | *Z. maize* | *GrTri14* | 214 | 24.34 | 8.74 | *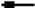* |
|  | *S. moellendorffii* | *SmTri13* | 373 | 41.78 | 7.66 | *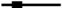* |
|  | *A. thaliana* | *AtTri11* | 372 | 42.78 | 5.68 | *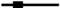* |
|  | *A. thaliana* | *AtTri12* | 406 | 46.68 | 6.38 | *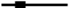* |
|  | *M. guttatus* | *MgTri11* | 396 | 44.33 | 5.68 | *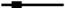* |
|  | *A. caerulea* | *AcTri07* | 382 | 43.46 | 5.89 | *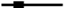* |
|  | *M. guttatus* | *MgTri12* | 326 | 37.38 | 5.73 | *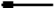* |
|  | *A. caerulea* | *AcTri08* | 407 | 43.21 | 5.83 | *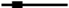* |
|  | *O. sativa* | *OsTri09* | 385 | 41.93 | 5.82 |  |
|  | *B. distachyon* | *BdTri08* | 379 | 41.40 | 5.96 |  |
|  | *S. bicolor* | *SbTri08* | 382 | 41.89 | 6.14 |  |
|  | *Z. maize* | *GrTri15* | 379 | 41.94 | 6.28 |  |
|  | *S. italic* | *SiTri10* | 385 | 42.30 | 6.36 |  |
|  | *S. italic* | *SiTri11* | 382 | 41.74 | 6.36 |  |
|  | *Z. maize* | *GrTri16* | 673 | 76.47 | 9.57 |  |

**Supplementary Table 4 Unrooted phylogenetic tree of trihelix gene members of clade III.**

| **Gene ID** | **Species** | **Name** | **Length** | **Mass** | **PI** | **1 200 400 600 (a.a)** |
| --- | --- | --- | --- | --- | --- | --- |
|  | *S. bicolor* | *SbTri09* | 448 | 50.69 | 6.08 |  |
|  | *Z. maize* | *GrTri17* | 447 | 50.51 | 6.11 |  |
|  | *S. italica* | *SiTri12* | 447 | 51.03 | 6.21 |  |
|  | *O. sativa* | *OsTri10* | 433 | 48.77 | 6.13 |  |
|  | *B. distachyon* | *BdTri09* | 430 | 49.11 | 5.76 |  |
|  | *S. bicolor* | *SbTri10* | 432 | 49.07 | 6.19 |  |
|  | *S. italica* | *SiTri13* | 435 | 49.67 | 6.12 |  |
|  | *Z. maize* | *GrTri18* | 437 | 49.78 | 6.47 |  |
|  | *O. sativa* | *OsTri11* | 484 | 55.06 | 6.24 |  |
|  | *T. aestivum* | *TaTri05* | 440 | 50.33 | 6.84 |  |
|  | *B. distachyon* | *BdTri10* | 423 | 47.83 | 7.01 |  |
|  | *O. sativa* | *OsTri12* | 411 | 46.67 | 6.28 |  |
|  | *B. distachyon* | *BdTri11* | 401 | 46.18 | 5.77 |  |
|  | *S. italica* | *SiTri14* | 403 | 45.22 | 6.17 |  |
|  | *S. bicolor* | *SbTri11* | 410 | 46.18 | 6.10 |  |
|  | *Z. maize* | *GrTri19* | 406 | 46.19 | 5.91 |  |
|  | *A. thaliana* | *AtTri13* | 386 | 44.96 | 6.59 |  |
|  | *M. guttatus* | *MgTri13* | 412 | 47.72 | 6,59 |  |
|  | *M. guttatus* | *MgTri14* | 385 | 44.75 | 6.14 |  |
|  | *A. caerulea* | *AcTri09* | 449 | 51.47 | 6.20 |  |
|  | *A. thaliana* | *AtTri14* | 444 | 50.93 | 5.88 |  |
|  | *M. guttatus* | *MgTri15* | 440 | 49.30 | 5.70 |  |
|  | *A. caerulea* | *AcTri10* | 378 | 43.56 | 9.24 |  |
|  | *A. caerulea* | *AcTri11* | 475 | 54.69 | 6.25 |  |
|  | *A. thaliana* | *AtTri15* | 432 | 48.87 | 8.03 |  |
|  | *B. distachyon* | *BdTri12* | 510 | 55.19 | 6.46 |  |
|  | *Z. maize* | *GrTri20* | 534 | 57.19 | 6.09 |  |
|  | *O. sativa* | *OsTri13* | 548 | 57.86 | 6.33 |  |
|  | *S. bicolor* | *SbTri12* | 542 | 58.12 | 6.25 |  |
|  | *Z. maize* | *GrTri21* | 529 | 56.70 | 7.38 |  |
|  | *M. guttatus* | *MgTri16* | 373 | 42.76 | 9.20 |  |
