# Supplementary material for: Evolutionary History of Trihelix Family and Their Functional Diversification
Source: DNA Res. 2014 May 25;21(5):499–510. doi: 10.1093/dnares/dsu016 (PMC4195496; doi:10.1093/dnares/dsu016)
Supplement: Supplementary Data [file supp_dsu016_dsu016supp_table5-6.doc]

**Supplementary Table 5 Unrooted phylogenetic tree of trihelix gene members of clade IV.**

| **Gene ID** | **Species** | **Name** | **Length** | **Mass** | **PI** | **1 200 400 600 (a.a)** |
| --- | --- | --- | --- | --- | --- | --- |
|  | *S. italica* | *SiTri15* | 327 | 35.38 | 8.99 | *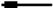* |
|  | *S. italica* | *SiTri16* | 327 | 35.38 | 8.99 | *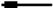* |
|  | *S. bicolor* | *SbTri13* | 338 | 36.73 | 5.89 | *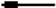* |
|  | *Z. maize* | *GrTri22* | 334 | 36.44 | 5.89 | *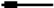* |
|  | *O. sativa* | *OsTri14* | 329 | 34.88 | 5.76 | *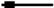* |
|  | *B. distachyon* | *BdTri13* | 326 | 35.24 | 6.30 | *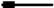* |
|  | *O. sativa* | *OsTri15* | 374 | 39.55 | 5.17 | *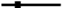* |
|  | *B. distachyon* | *BdTri14* | 364 | 39.25 | 5.09 | *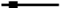* |
|  | *S. italica* | *SiTri17* | 348 | 37.41 | 5.22 | *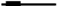* |
|  | *Z. maize* | *GrTri23* | 340 | 36.76 | 5.27 | *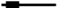* |
|  | *Z. maize* | *GrTri24* | 366 | 38.95 | 5.01 | *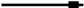* |
|  | *T. aestivum* | *TaTri06* | 259 | 27.53 | 7.73 | *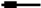* |
|  | *A. caerulea* | *AcTri12* | 331 | 37.29 | 5.70 | *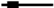* |
|  | *A. caerulea* | *AcTri13* | 312 | 35.49 | 5.73 | *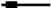* |
|  | *M. guttatus* | *MgTri17* | 335 | 36.65 | 5.45 | *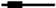* |
|  | *A. thaliana* | *AtTri16* | 314 | 34.86 | 6.01 | *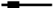* |
|  | *M. guttatus* | *MgTri18* | 284 | 32.04 | 6.63 | *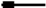* |
|  | *O. sativa* | *OsTri16* | 298 | 31.55 | 7.76 | *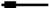* |
|  | *S. bicolor* | *SbTri14* | 300 | 31.73 | 8.93 | *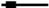* |
|  | *Z. maize* | *GrTri25* | 277 | 30.18 | 8.94 | *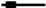* |
|  | *S. italica* | *SiTri18* | 287 | 31.87 | 9.25 | *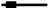* |
|  | *S. italica* | *SiTri19* | 287 | 30.95 | 9.90 | *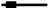* |
|  | *P. patens* | *PpTri12* | 319 | 35.72 | 6.20 | *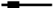* |
|  | *P. patens* | *PpTri13* | 798 | 87.31 | 8.49 | *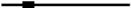* |
|  | *P. patens* | *PpTri14* | 667 | 72.38 | 7.34 | 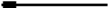 |
|  | *A. thaliana* | *AtTri17* | 294 | 33.37 | 4.87 | 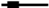 |
|  | *A. caerulea* | *AcTri14* | 255 | 29.40 | 8.57 | 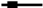 |
|  | *S. italica* | *SiTri20* | 278 | 30.00 | 4.31 | 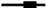 |
|  | *B. distachyon* | *BdTri15* | 367 | 38.37 | 4.23 | 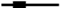 |
|  | *O. sativa* | *OsTri17* | 435 | 46.24 | 4.45 | 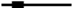 |
|  | *S. bicolor* | *SbTri15* | 447 | 47.02 | 4.60 | 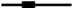 |
|  | *Z. maize* | *GrTri26* | 387 | 40.51 | 4.55 | 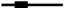 |
|  | *S. moellendorffii* | *SmTri06* | 299 | 32.75 | 9.11 | 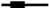 |
|  | *S. moellendorffii* | *SmTri07* | 276 | 30.39 | 6.56 | 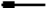 |
|  | *P. patens* | *PpTri15* | 311 | 35.44 | 7.01 | 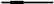 |
|  | *S. moellendorffii* | *SmTri08* | 388 | 43.20 | 8.59 | 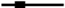 |
|  | *P. patens* | *PpTri16* | 333 | 37.88 | 5.95 | 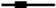 |
|  | *P. patens* | *PpTri17* | 333 | 47.85 | 5.84 | 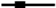 |
|  | *P. patens* | *PpTri18* | 334 | 37.67 | 6.11 | 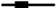 |
|  | *P. patens* | *PpTri19* | 319 | 36.54 | 8.78 | 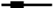 |
|  | *P. patens* | *PpTri20* | 333 | 37.88 | 5.95 | 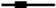 |
|  | *P. patens* | *PpTri21* | 309 | 35.17 | 8.41 | 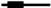 |
|  | *P. patens* | *PpTri22* | 700 | 78.52 | 6.02 | 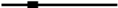 |
|  | *P. patens* | *PpTri23* | 280 | 32.10 | 4.46 | 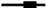 |
|  | *P. patens* | *PpTri24* | 1070 | 120.79 | 5.21 | 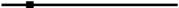 |
|  | *S. bicolor* | *SbTri16* | 360 | 39.49 | 6.07 | 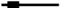 |
|  | *S. italica* | *SiTri21* | 367 | 39.40 | 6.96 | 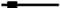 |
|  | *B. distachyon* | *BdTri16* | 341 | 37.20 | 8.68 | 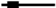 |
|  | *Z. maize* | *GrTri27* | 232 | 25.83 | 9.72 | 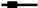 |
|  | *O. sativa* | *OsTri18* | 345 | 36.84 | 6.16 | 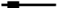 |
|  | *A. caerulea* | *AcTri15* | 263 | 29.86 | 8.77 | 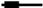 |
|  | *A. thaliana* | *AtTri18* | 383 | 42.59 | 9.13 | 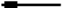 |
|  | *A. thaliana* | *AtTri19* | 340 | 38.31 | 9.03 | 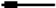 |
|  | *M. guttatus* | *MgTri19* | 356 | 39.63 | 9.40 | 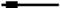 |
|  | *S. moellendorffii* | *SmTri09* | 263 | 32.13 | 8.76 | 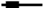 |
|  | *S. moellendorffii* | *SmTri10* | 397 | 44.03 | 9.02 | 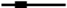 |
|  | *A. caerulea* | *AcTri16* | 361 | 40.72 | 9.25 | 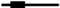 |
|  | *B. distachyon* | *BdTri17* | 411 | 44.26 | 10.05 | 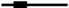 |
|  | *O. sativa* | *OsTri19* | 390 | 41.44 | 9.01 | 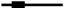 |
|  | *S. bicolor* | *SbTri17* | 374 | 40.20 | 9.61 | 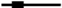 |
|  | *Z. maize* | *GrTri28* | 381 | 40.87 | 9.38 | 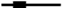 |
|  |  |  |  |  |  |  |

**Supplementary Table 6 Unrooted phylogenetic tree of trihelix gene members of clade V.**

| **Gene ID** | **Species** | **Name** | **Length** | **Mass** | **PI** | **1 200 400 600 (a.a)** |
| --- | --- | --- | --- | --- | --- | --- |
|  | *S. italica* | *SiTri22* | 395 | 42.29 | 7.86 | *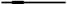* |
|  | *S. italica* | *SiTri23* | 395 | 43.21 | 8.53 | *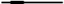* |
|  | *S. bicolor* | *SbTri18* | 419 | 44.59 | 8.55 | *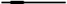* |
|  | *Z. maize* | *GrTri29* | 402 | 42.97 | 7.15 | *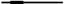* |
|  | *Z. maize* | *GrTri30* | 682 | 72.41 | 9.61 | *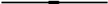* |
|  | *B. distachyon* | *BdTri18* | 413 | 44.22 | 6.56 | *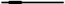* |
|  | *O. sativa* | *OsTri20* | 419 | 44.44 | 6.11 | *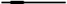* |
|  | *Z. maize* | *GrTri31* | 439 | 46.48 | 8.87 | *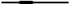* |
|  | *B. distachyon* | *BdTri19* | 418 | 45.34 | 6.97 | *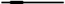* |
|  | *S. italica* | *SiTri24* | 405 | 42.74 | 6.13 | *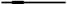* |
|  | *S. italica* | *SiTri25* | 405 | 44.21 | 6.58 | *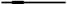* |
|  | *O. sativa* | *OsTri21* | 418 | 43.93 | 7.04 | *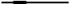* |
|  | *S. bicolor* | *SbTri19* | 408 | 43.14 | 6.24 | *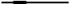* |
|  | *Z. maize* | *GrTri32* | 392 | 42.11 | 6.61 | *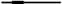* |
|  | *A. thaliana* | *AtTri20* | 443 | 48.31 | 9.63 | *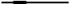* |
|  | *A. caerulea* | *AcTri17* | 359 | 40.04 | 9.75 | *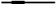* |
|  | *S. moellendorffii* | *SmTri14* | 222 | 24.80 | 9.82 | *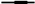* |
|  | *S. moellendorffii* | *SmTri15* | 226 | 25.49 | 9.74 | *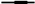* |
|  | *A. thaliana* | *AtTri21* | 249 | 28.39 | 5.38 | *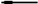* |
|  | *A. thaliana* | *AtTri22* | 249 | 28.56 | 8.67 | *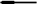* |
|  | *A. caerulea* | *AcTri18* | 300 | 34.59 | 9.21 | *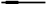* |
|  | *A. thaliana* | *AtTri23* | 321 | 36.05 | 9.48 | *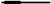* |
|  | *A. caerulea* | *AcTri19* | 256 | 29.68 | 9.48 | *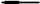* |
|  | *M. guttatus* | *MgTri20* | 375 | 42.74 | 8.26 | *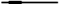* |
|  | *A. caerulea* | *AcTri20* | 316 | 35.65 | 9.46 | *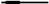* |
|  | *M. guttatus* | *MgTri21* | 317 | 35.78 | 5.65 | *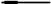* |
|  | *M. guttatus* | *MgTri22* | 306 | 34.34 | 5.67 | *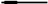* |
|  | *S. bicolor* | *SbTri20* | 347 | 37.16 | 9.89 | *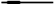* |
|  | *Z. maize* | *GrTri33* | 343 | 36.92 | 9.91 | *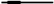* |
|  | *B. distachyon* | *BdTri20* | 353 | 37.95 | 9.85 | *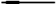* |
|  | *O. sativa* | *OsTri22* | 346 | 37.44 | 9.92 | *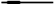* |
|  | *S. italica* | *SiTri26* | 343 | 37.07 | 9.91 | *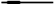* |
|  | *S. italica* | *SiTri27* | 343 | 37.07 | 9.91 | *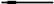* |
|  | *S. italica* | *SiTri28* | 318 | 34.58 | 9.84 | *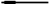* |
|  | *B. distachyon* | *BdTri21* | 320 | 35.03 | 9.80 | *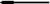* |
|  | *O. sativa* | *OsTri23* | 332 | 35.92 | 9.62 | *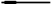* |
|  | *S. italica* | *SiTri29* | 324 | 35.14 | 9.78 | *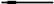* |
|  | *S. bicolor* | *SbTri21* | 317 | 34.64 | 9.67 | *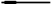* |
|  | *Z. maize* | *GrTri34* | 318 | 34.72 | 9.59 | *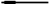* |
|  | *Z. maize* | *GrTri35* | 315 | 34.67 | 9.87 |  |
|  | *A. thaliana* | *AtTri24* | 383 | 41.73 | 8.73 |  |
|  | *Z. maize* | *GrTri36* | 210 | 23.06 | 10.02 |  |
|  | *M. guttatus* | *MgTri23* | 376 | 42.61 | 4.61 |  |
|  | *A. caerulea* | *AcTri21* | 419 | 48.17 | 4.51 |  |
|  | *A. caerulea* | *AcTri22* | 294 | 34.35 | 4.75 |  |
|  | *A. thaliana* | *AtTri25* | 333 | 39.08 | 4.65 |  |
|  | *M. guttatus* | *MgTri24* | 322 | 37.08 | 4.59 |  |
|  | *B. distachyon* | *BdTri22* | 303 | 33.82 | 6.57 |  |
|  | *O. sativa* | *OsTri24* | 322 | 35.76 | 5.28 |  |
|  | *S. italica* | *SiTri30* | 353 | 23.71 | 9.67 |  |
|  | *S. bicolor* | *SbTri22* | 429 | 47.57 | 8.69 |  |
|  | *Z. maize* | *GrTri37* | 350 | 38.80 | 9.70 |  |
|  | *S. italica* | *SiTri31* | 212 | 23.45 | 10.65 |  |
|  | *O. sativa* | *OsTri25* | 214 | 23.67 | 9.71 |  |
|  | *Z. maize* | *GrTri38* | 251 | 27.11 | 6.27 |  |
|  | *Z. maize* | *GrTri39* | 257 | 27.75 | 9.89 |  |
|  | *O. sativa* | *OsTri26* | 255 | 27.44 | 9.75 |  |
|  | *B. distachyon* | *BdTri23* | 259 | 27.84 | 9.65 |  |
|  | *S. moellendorffii* | *SmTri16* | 725 | 80.39 | 6.68 |  |
|  | *S. moellendorffii* | *SmTri17* | 626 | 68.48 | 6.63 |  |
|  | *Z. maize* | *GrTri40* | 337 | 36.72 | 6.94 |  |
|  | *S. italica* | *SiTri32* | 337 | 36.22 | 8.25 |  |
|  | *B. distachyon* | *BdTri24* | 344 | 36.98 | 7.56 |  |
|  | *Z. maize* | *GrTri41* | 1021 | 110.24 | 5.96 |  |
|  | *S. bicolor* | *SbTri23* | 337 | 36.56 | 8.20 |  |
|  | *O. sativa* | *OsTri27* | 336 | 36.34 | 6.58 |  |
|  | *A. thaliana* | *AtTri26* | 296 | 33.28 | 9.43 |  |
|  | *M. guttatus* | *MgTri25* | 368 | 40.81 | 9.29 |  |
|  | *A. caerulea* | *AcTri23* | 329 | 36.76 | 9.37 |  |
|  | *P. patens* | *PpTri25* | 342 | 38.04 | 8.51 |  |
|  | *S. moellendorffii* | *SmTri18* | 362 | 38.88 | 8.19 |  |
|  | *S. moellendorffii* | *SmTri19* | 809 | 86.72 | 9.02 |  |
|  | *S. bicolor* | *SbTri24* | 227 | 24.81 | 11.19 |  |
|  | *Z. maize* | *GrTri42* | 206 | 22.63 | 11.19 |  |
|  | *Z. maize* | *GrTri43* | 208 | 22.95 | 11.09 |  |
|  | *O. sativa* | *OsTri28* | 205 | 22.37 | 10.08 |  |
|  | *B. distachyon* | *BdTri25* | 128 | 14.01 | 11.03 |  |
|  | *S. bicolor* | *SbTri25* | 316 | 35.39 | 5.55 |  |
|  | *Z. maize* | *GrTri44* | 319 | 35.88 | 5.60 |  |
|  | *Z. maize* | *GrTri45* | 292 | 32.92 | 7.79 |  |
|  | *S. italica* | *SiTri33* | 331 | 36.72 | 5.37 |  |
|  | *O. sativa* | *OsTri29* | 315 | 35.06 | 7.09 |  |
|  | *B. distachyon* | *BdTri26* | 329 | 36.44 | 5.69 |  |
|  | *T. aestivum* | *TaTri07* | 308 | 35.06 | 5.34 |  |
|  | *A. caerulea* | *AcTri24* | 332 | 38.00 | 5.53 |  |
|  | *M. guttatus* | *MgTri26* | 345 | 39.27 | 8.96 |  |
|  | *A. thaliana* | *AtTri27* | 310 | 35.20 | 9.23 |  |
|  | *A. thaliana* | *AtTri28* | 372 | 40.78 | 9.08 |  |
|  | *M. guttatus* | *MgTri27* | 293 | 32.96 | 8.61 |  |
|  | *A. caerulea* | *AcTri25* | 403 | 44.01 | 9.28 |  |
|  | *A. thaliana* | *AtTri29* | 310 | 34.43 | 5.75 |  |
|  | *S. italica* | *SiTri34* | 317 | 34.11 | 9.41 |  |
|  | *S. bicolor* | *SbTri26* | 322 | 34.27 | 9.48 |  |
|  | *Z. maize* | *GrTri46* | 321 | 34.10 | 9.42 |  |
|  | *B. distachyon* | *BdTri27* | 324 | 34.30 | 9.53 |  |
|  | *Z. maize* | *GrTri47* | 318 | 33.60 | 9.17 |  |
|  | *T. aestivum* | *TaTri08* | 317 | 33.62 | 9.53 |  |
|  | *O. sativa* | *OsTri30* | 336 | 35.64 | 8.90 |  |
|  | *S. moellendorffii* | *SmTri20* | 532 | 60.00 | 8.53 |  |
|  | *S. moellendorffii* | *SmTri21* | 487 | 54.63 | 5.97 |  |
|  | *P. patens* | *PpTri26* | 430 | 48.06 | 6.22 |  |
|  | *P. patens* | *PpTri27* | 414 | 46.58 | 6.24 |  |
|  | *P. patens* | *PpTri28* | 433 | 48.28 | 6.66 |  |
|  | *P. patens* | *PpTri29* | 407 | 45.87 | 6.15 |  |
|  | *S. moellendorffii* | *SmTri22* | 402 | 42.08 | 9.41 |  |
|  | *S. moellendorffii* | *SmTri23* | 392 | 43.43 | 9.39 |  |
|  | *S. moellendorffii* | *SmTri24* | 392 | 43.43 | 9.39 |  |
|  | *A. caerulea* | *AcTri26* | 508 | 56.14 | 8.38 |  |
|  | *A. caerulea* | *AcTri27* | 570 | 62.63 | 6.79 |  |
|  | *A. thaliana* | *AtTri30* | 542 | 59.25 | 6.12 |  |
|  | *M. guttatus* | *MgTri28* | 473 | 51.46 | 6.51 |  |
|  | *B. distachyon* | *BdTri28* | 502 | 53.76 | 7.53 |  |
|  | *S. italica* | *SiTri35* | 473 | 50.13 | 8.73 |  |
|  | *S. italica* | *SiTri36* | 437 | 46.30 | 8.38 |  |
|  | *S. bicolor* | *SbTri27* | 519 | 55.26 | 7.16 |  |
|  | *Z. maize* | *GrTri48* | 519 | 55.33 | 7.19 |  |
|  | *S. italica* | *SiTri37* | 536 | 57.35 | 6.55 |  |
|  | *S. italica* | *SiTri38* | 519 | 55.12 | 6.84 |  |
|  | *S. italica* | *SiTri39* | 497 | 52.94 | 7.18 |  |
|  | *S. moellendorffii* | *SmTri25* | 1045 | 114.53 | 6.19 |  |
